# Elasticity of the HIV-1 core facilitates nuclear entry and infection

**DOI:** 10.1371/journal.ppat.1012537

**Published:** 2024-09-11

**Authors:** Akshay Deshpande, Alexander J. Bryer, Jonathan R. Andino-Moncada, Jiong Shi, Jun Hong, Cameron Torres, Shimon Harel, Ashwanth C. Francis, Juan R. Perilla, Christopher Aiken, Itay Rousso

**Affiliations:** 1 Ben-Gurion University of the Negev, Department of Physiology and Cell Biology, Beer Sheva, Israel; 2 University of Delaware, Department of Chemistry and Biochemistry, Newark, Delaware, United States of America; 3 Florida State University, Institute of Molecular Biophysics, Tallahassee, Florida, United States of America; 4 Vanderbilt University Medical Center, Department of Pathology, Microbiology and Immunology, Nashville, Tennessee, United States of America; 5 Florida State University, Department of Biological Sciences, Tallahassee, Florida, United States of America; Loyola University Chicago, UNITED STATES OF AMERICA

## Abstract

HIV-1 infection requires passage of the viral core through the nuclear pore of the cell, a process that depends on functions of the viral capsid. Recent studies have shown that HIV-1 cores enter the nucleus prior to capsid disassembly. Interactions of the viral capsid with the nuclear pore complex are necessary but not sufficient for nuclear entry, and the mechanism by which the viral core traverses the comparably sized nuclear pore is unknown. Here we show that the HIV-1 core is highly elastic and that this property is linked to nuclear entry and infectivity. Using atomic force microscopy-based approaches, we found that purified wild type cores rapidly returned to their normal conical morphology following a severe compression. Results from independently performed molecular dynamic simulations of the mature HIV-1 capsid also revealed its elastic property. Analysis of four HIV-1 capsid mutants that exhibit impaired nuclear entry revealed that the mutant viral cores are brittle. Adaptation of two of the mutant viruses in cell culture resulted in additional substitutions that restored elasticity and rescued infectivity and nuclear entry. We also show that capsid-targeting compound PF74 and the antiviral drug Lenacapavir reduce core elasticity and block HIV-1 nuclear entry at concentrations that preserve interactions between the viral core and the nuclear envelope. Our results indicate that elasticity is a fundamental property of the HIV-1 core that enables nuclear entry, thereby facilitating infection. These results provide new insights into the role of the capsid in HIV-1 nuclear entry and the antiviral mechanisms of HIV-1 capsid inhibitors.

## Author summary

The genetic material of HIV-1 is packaged inside a cone shaped container that is called the viral core. To infect a cell, the viral core must pass through the cell’s nuclear pore. Using atomic force microscopy, a technique that uses a tiny needle to feel the surface of things at the microscopic level, and molecular dynamic simulations, we discovered that wild-type HIV-1 cores quickly revert to their normal shape after compression, while capsid mutants with impaired nuclear entry are brittle. Restoring elasticity in these mutants recovered their ability to infect. Treatment with capsid-targeting drugs also reduced core elasticity. These findings suggest that capsid elasticity is essential for HIV-1 nuclear entry and infection, offering new insights into HIV-1 mechanics and potential antiviral strategies.

## Introduction

The HIV-1 capsid, which forms the shell of the core and acts as a protective barrier, encapsidates the enzymes reverse transcriptase and integrase and the viral genomic RNA. Following delivery of the core into the target cell cytoplasm, the HIV-1 genome is reverse-transcribed into double-stranded DNA and is transported into the nucleus, where it undergoes integration into the host chromosomal DNA. The viral capsid plays a critical role in HIV-1 nuclear entry. Docking of the HIV-1 core at the nuclear pore complex (NPC) and subsequent nuclear entry are promoted by interactions between the capsid and cellular factors, including cyclophilin A, CPSF6, and nucleoporins (e.g. Nup358 and Nup153) [1–9]. Recent studies suggest that the core is intact during nuclear entry, thereby ensuring completion of reverse transcription within the nucleus [10,11]. However, it is presently unclear how the core passes through the comparably sized nuclear pore [2,12,13] while remaining intact.

The HIV-1 capsid consists of a lattice of CA protein hexamers and pentamers in the shape of a cone. The biological and mechanical stability of the capsid are critical for infection. Treatment with low concentrations of the capsid-binding compounds PF-3450074 (PF74) [14] and Lenacapavir (LCV) [15] reduces HIV-1 infectivity by inhibiting nuclear entry [16–24]. Nuclear entry can also be reduced by mutations in the capsid protein (CA). For example, the capsid-stabilizing CA mutation E45A markedly reduces nuclear entry and consequently infectivity [25,26]. However, position 45 in CA resides at an inter-subunit interface in the capsid that is not a binding site for known host proteins that promote nuclear entry [27]. In addition, E45 lies outside the binding site for PF74 and LCV, yet the mutant virus is resistant to these compounds [17,28]. It is noteworthy that the suppressor mutation R132T rescues nuclear entry and infectivity of the E45A mutant and restores its sensitivity to PF74 without reversing the hyperstability of the capsid [26]. These observations suggested that a property of the capsid distinct from stability is crucial for facilitating nuclear entry of the core.

Here we show that the native HIV-1 core is highly elastic, a property that enables it to withstand extreme deformation without experiencing detectable structural failure. This unusual core resilience is reduced by perturbations that impair nuclear entry, including the E45A substitution in CA and treatment with PF74 and LCV. Acquisition of suppressor mutations restored elasticity to the mutant cores, leading to the rescue of nuclear entry and the improved infectivity of the mutant virions. These results uncover elasticity as a mechanical property of the HIV-1 core that is essential for nuclear entry.

## Results

### Wild type HIV-1 cores are highly elastic

**Fig 1:**
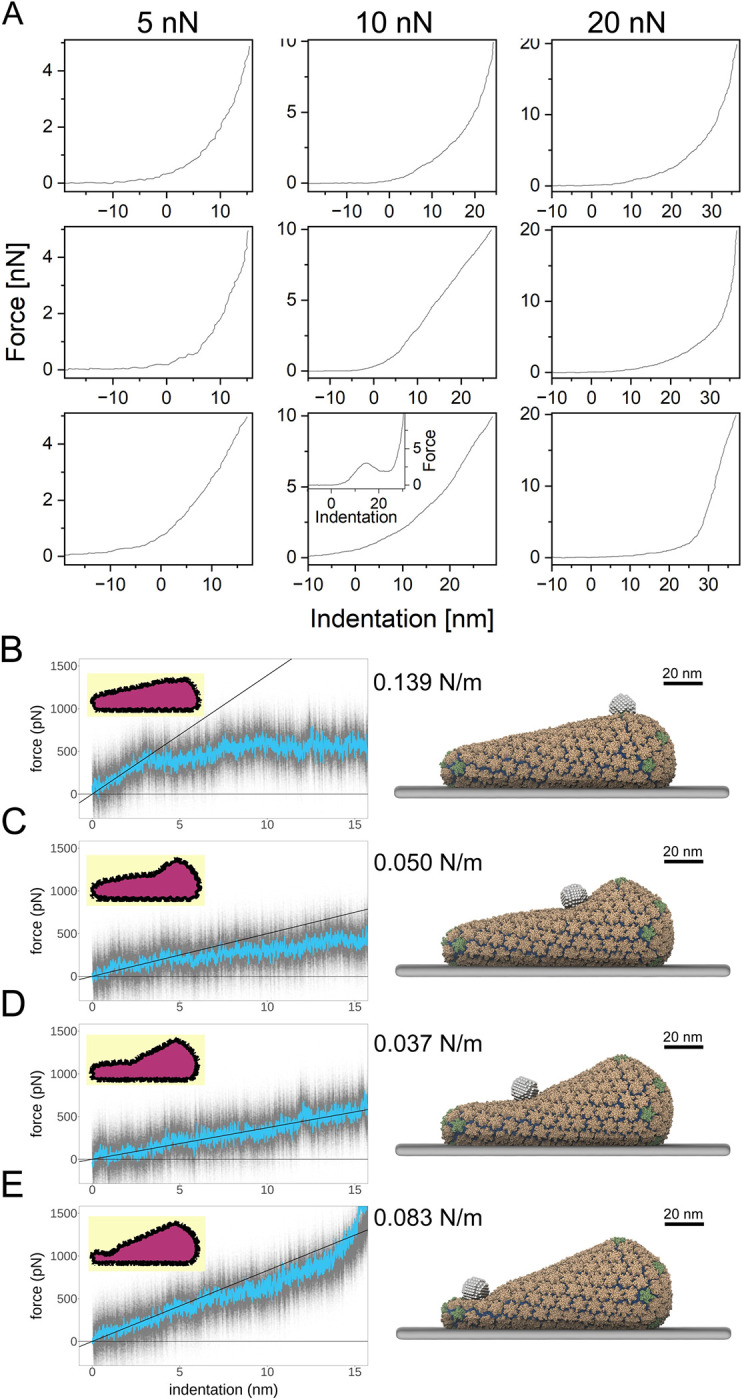
Experimental and simulated AFM nanoindentation of wild type IP6-treated cores. (A) Representative force–distance (FD) curves where force loading curves are shown. The maximal loading force was set to 5 nN (*n* = 23), 10 nN (*n* = 24), and 20 nN (*n* = 25). Despite the application of high maximal loading forces, none of the curves exhibited any indication of structural failure in the core. In one core (of the 72 examined), we observed an abrupt drop in the loading curve (inset). (B-E) Simulated FD curves for nanoindentation of coarse-grained HIV-1 capsids, testing the effect of probe location, ultimately capsid curvature, on measured material properties. The plots show raw data in gray, and a windowed-average in blue. The inset volume cross-sections show the deformed capsid morphologies and provide a sense of global deformation. The solid black line shows a linear fit of the FD curve for the first 3 nm of indentation, which was used to derive the stiffness values given to the right of each plot. Additionally, snapshots from these nanoindentation simulations are provided on the right, with all components visualized: spherical AFM probes, capsids, and baseplates. (B) Probe located at the large end of the conical capsid. Note the global deformation, narrowing, of the cone in the inset graphic. (C) and (D) show probe locations in the relatively flat, mid-regions of the capsid. (E) Shows nanoindentation of the narrow, most highly curved region of the capsid.

Studies of the HIV-1 capsid have demonstrated that the inter-subunit interfaces are structurally variable, indicating that the capsid is flexible [29–31]. In previous studies, we employed Atomic Force Microscopy (AFM) to measure the stiffness of purified HIV-1 cores, defined as the resistance to deformation by an applied force. We identified substitutions in CA that altered the stiffness of cores, and showed that the cores exhibit abrupt changes in stiffness during reverse transcription *in vitro* [32,33]. Another property that can be associated with solid materials is elasticity, which is defined as the degree to which a material is restored to its initial shape without breaking following a physical perturbation. In an effort to understand the nuclear entry defect associated with the E45A mutant, we sought to examine the potential role of capsid elasticity in nuclear entry. To quantify elasticity, we exploited the ability of the AFM probe to induce structural deformation in the viral core and analyzed the consequences on the mechanical behavior and structural integrity of the capsid. For this purpose, the AFM was operated in the nano-indentation mode, but with high applied forces (≥ 5 nN, vs. the 1–1.5 nN range used in measurements of core stiffness) to explore the mechanical tolerance limits of the cores and quantify their critical loading force: the force at which there is an abrupt drop in the force-distance curve, representing the failure and collapse of the structure [34]. With wild type cores, we observed no evidence for structural failure in the force–distance curves, even at a maximal loading force of 20 nN (Fig 1A). Morphological analysis by scanning AFM further demonstrated that all cores (n = 72) showed no evidence for structural damage following perturbation (S1 Fig). For a single core in the 10 nN applied force group, we observed a drop in the force–distance curve (Fig 1A inset). Morphological analysis of this core structure showed that it remained intact and non-deformed. Therefore, this core likely underwent reversible buckling rather than breakage. The results show that wild type HIV-1 cores can withstand marked deformation without undergoing irreversible structural damage, indicating that the HIV-1 capsid is highly elastic. This finding is in sharp contrast to other viruses, such as bacteriophage HK97, hepatitis B virus, and herpes simplex virus, which have brittle capsids that undergo structural failure at forces as low as 1–6 nN [35].

### In silico modeling supports the elasticity of the HIV-1 capsid

To complement the AFM measurements, we performed coarse grained *in silico* computational nanoindentation simulations at four distinct regions along the wild-type capsid surface to model the resistance of the capsid to deformation. After placing four spherical AFM probes equidistant along the capsid’s surface (Fig 1B–1E), we modeled a constant pushing velocity to the tip (3.125 nm/μs). While the simulated tip velocity is higher than what is achievable in physical experiments, simulations can provide a high temporal and spatial resolution view of how the capsid deforms and responds to mechanical stress [36–39]. Indentation of the broader region of the capsid initially showed the highest stiffness among the four probed regions, with the capsid appearing to buckle [40–42] after 3 nm of indentation demonstrating a change in the elasticity of the capsid’s mechanical properties at indentation depths beyond 3 nm (Fig 1B). Indentations in the relatively flat, middle regions of the capsid revealed the lowest stiffness (Fig 1C and 1D) and the most linearity in FD-curves. The narrow end of the capsid showed high stiffness through the indentation profile, until contact with the baseplate is made (Fig 1E). Our simulations are consistent with the experimental AFM data and show that the wild-type capsid is a robust and elastic container, able to withstand extreme deformations and recover (S2 Fig) prior to failure of the intermolecular interfaces comprising the CA lattice. The different stiffness values observed at different positions further suggest that the HIV-1 capsid has a material heterogeneity that is determined by its variable curvature.

### WT HIV-1 cores can reversibly undergo large compression

**Fig 2:**
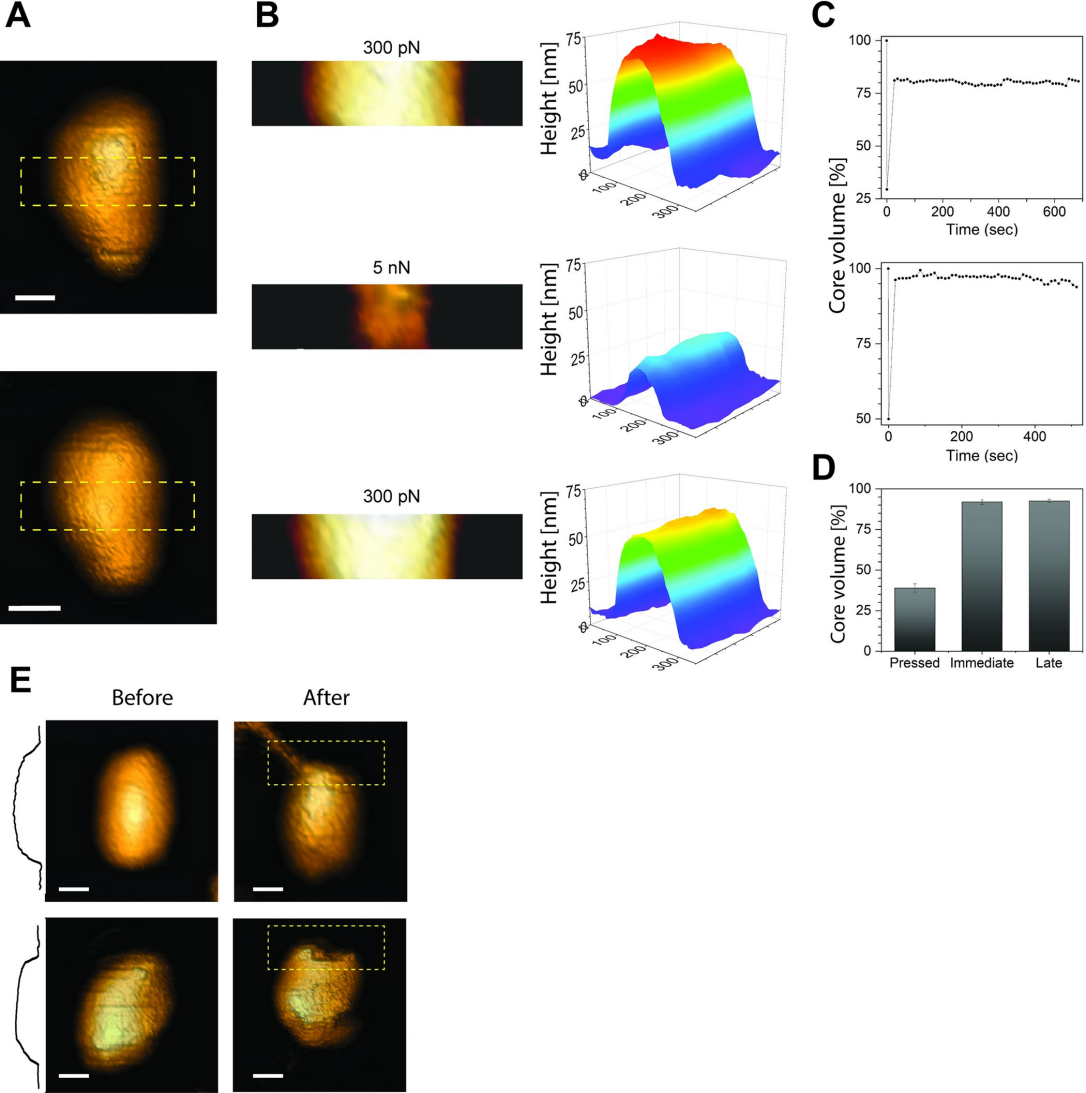
Volume recovery of the HIV core following compression. (A) Topographic AFM images of an isolated IP6-treated WT core before (upper image) and after (lower image) extreme compression. Both images were acquired using the QI mode at a maximal loading force of 300 pN. Subsequent measurements focused on a selected central region, as marked by a dashed yellow rectangle. It should be noted that the length of the core appears to be larger than expected due to convolution between the AFM tip and the core. Scale bars are 50 nm. (B) Representative topographic AFM images (left panels) and the corresponding Matlab plots (right panels) for the selected central region of an isolated IP6-treated WT core before (top), during (middle), and immediately after (bottom) compression by a 5 nN loading force (the before and after image were acquired at 300 pN maximal loading force). The same region was repeatedly scanned and imaged over time in QI mode at maximal loading force of 300 pN. The reduced height during imaging at 5 nN is higher than the observed indentation at the same force (as shown in Fig 1) is the result of the different AFM modes used to acquired the data. In the imaging mode, a few dozen of FD curves are rapidly applied to the region whereas Fig 1 shows a single FD curve. (C) Two representative volume recovery trajectories (of the 36 trajectories obtained). The volume beneath the selected region was calculated using MatLab and plotted as a function of time. The initial volume was set as 100%, and all other measurements were normalized accordingly. The upper curve shows nearly full volume recovery, whereas the lower curve shows 80% recovery, which was the minimum volume recovery percentage we detected. (D) The average volume recovery of the 36 analyzed cores. The average compressed volume was 39% (pressed). The average volume upon reducing the force back to 300 pN was 92% (immediate). Several minutes (5–7 min) later, the recovery volume remained unchanged at 92% (late). T-test analysis revealed that the difference between the compressed and immediate or late recovered volume is significant (p value <0.0001). Error bars represent the standard error of the mean. (E) Topographic AFM images of two representative WT cores before and after compression. Images were acquired using the QI mode operated at a 300 pN loading force. Typical cone-shaped cores are observed. A cross-section height profile along the length of the core is displayed. After compression, most cores (~86%) remained intact. However, those that broke exhibited an opening at the end of the core, distal from the compressed region. For clarity, openings in the cores are shown within a dashed yellow rectangle. Scale bars are 50 nm.

To physically assess the elasticity of HIV-1 cores, we employed AFM to analyze the shape of the viral core soon after release of strong applied force. In this approach, a core was first imaged by scanning at a low loading force of 300 pN (Fig 2A). We then selected a region of interest (S3 Fig) located in the main body of the core and rescanned it. This region was then scanned at a high loading force (5 nN) that caused the core to compress dramatically at this region. The force was immediately reduced to 300 pN and the compressed region rapidly imaged repeatedly over time (Fig 2B). The volume under the scanned region was then calculated as a function of time. Fig 2C shows two representative trajectories in which the volume was compressed to 50% and 30% of the initial volume and recovered to nearly 100% and 80%, respectively. Mean values of the compressed volume (pressed: 39 ± 3%), the immediate recovery volume (immediate: 92 ± 1%), and the volume 5–7 minutes after compressing the core (later: 92 ± 1%) were determined from analysis of 36 individual cores (Fig 2D). This shows that wild type HIV-1 cores displayed nearly complete recovery after being strongly compressed. The remaining 8% of non-recovered volume suggest that the core undergo a small and partially plastic (irreversible) deformation. The measured recovery volume was highly consistent in nearly all the cores examined. Consequently, the averaged immediate and late volumes were identical, indicating that the core recovered its initial shape in a time shorter than the temporal resolution of the assay (10–15 seconds) (Fig 2C and 2D). These results further demonstrate that HIV-1 cores are highly elastic and rapidly recover nearly their full initial volume after being strongly compressed. For the majority of cores, we observed no structural damage in the compressed region of the capsid lattice. In 14% of cores analyzed, (5 of 36), structural damage was apparent near the end of the core following compression and recovery (Fig 2E). We previously reported that capsid disassembly begins at the end of isolated cores during *in vitro* reverse transcription [43]. This highly curved and pentamer-rich section has been proposed to be the least stable region in the HIV-1 capsid [22,43–47]. Our data are consistent with the aftermentioned model, in which the weakest region in the structure collapses as the core is compressed.

### HIV-1 CA mutants with nuclear entry defects contain brittle cores

**Fig 3:**
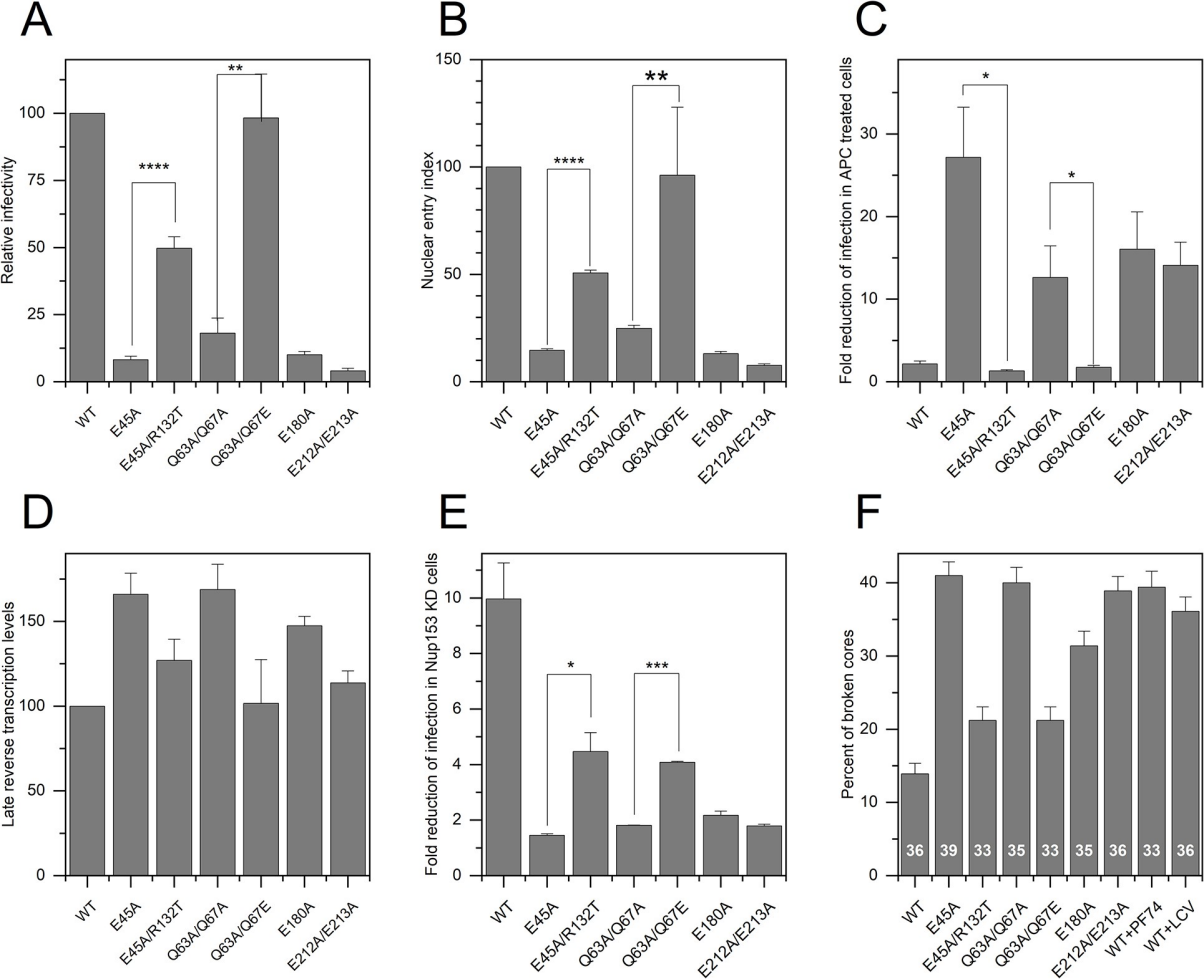
DNA synthesis, nuclear entry, infectivity and core brittleness of isolated HIV-1 cores. (A) Relative single-cycle infectivity of wild type and CA mutant viruses. (B) Nuclear entry efficiency of CA mutants relative to WT HIV-1. Values represent the ratio of 2-LTR circle levels to late reverse transcripts. (C) Ratio of infection of control Hela cells vs. aphidicolin-treated cells by WT and mutant viruses. (D) Relative levels of late reverse transcripts in target cells. (E) Fold reduction of infection in Nup153-depleted cells vs. control cells. For B-E, values shown are the mean of three independent experiments, with error bars representing the standard error of the mean. Error bars in all panels represent the standard error of the mean. (F) The percentage of broken wild-type (WT) and mutant cores. Cores were examined in the presence of IP6 (100 μM). WT cores were also treated with the capsid-inhibiting antiviral drugs PF74 (1.25 μM) or Lenacapavir (LCV; 500 pM). The number of cores analyzed is indicated inside each bar. Core breakage was determined by AFM imaging of the cores after they were compressed by a loading force of 5 nN applied during imaging of the middle section of the capsid (as in Fig 2). Error bars represent standard error of the mean. The error of the mean in (F) was obtain by running a bootstrap analysis. Representative AFM images of intact and broken cores following compression are shown in S12 Fig.

To investigate the potential role of elasticity in HIV-1 infection, we measured the infectivitry, nuclear entry, and reverse transcription efficiency of WT and a series of CA mutants HIV-1 cores (Fig 3). Like E45A, three additional CA mutants, exhibited low infectivity, impaired nuclear entry, and cell-cycle-dependent infection yet underwent efficient reverse transcription in target cells. Moreover, infection by these mutants was minimally affected by depletion of the nucleoporin Nup153, which reduces infection by wild type HIV-1 (Fig 3E) [48,49]. As previously reported, the suppressor mutation R132T partially rescued E45A infectivity and nuclear entry. Similarly, the pseudorevertant Q63A/Q67E, which emerged during prolonged culture of the Q63A/Q67A mutant in T cells, exhibited nuclear entry and infectivity comparable to the wild type (Fig 3).

We next measured the structural recovery of cores from CA mutants that exhibit impaired nuclear entry and quantified the percentage of broken cores following compression as a measure of inelasticity/brittleness (Fig 3F). Relative to the wild type, E45A mutant cores were more brittle (41% of cores were broken following compression). By contrast, E45A/R132T cores were less brittle, exhibiting a frequency of breakage (21%) closer to that of wild type cores (14%), indicating that the addition of the suppressor mutation restored elasticity to the E45A mutant capsid. Simulated nanoindentation of E45A and E45A/R132T capsids also showed that the mutations produced a significant increase in stiffness (S4 Fig) and, additionally, affected capsid deformability (S4–S7 Figs), with E45A capsids in particular rupturing at relatively smaller indentation distances compared to wild type and E45A/R132T while absorbing more energy through deformation. Importantly, when we considered simulated probe locations, and thus regions of the capsid, independently, we found that wild type, E45A and E45A/R132T capsids have similar stiffness in the low curvature mid-regions of the capsid (S8 Fig). At the highly curved broad end, the wild type was slightly stiffer than either mutant, further demonstrating that the capsid’s material response is predicated by its shape and location of indentation.

**Fig 4:**
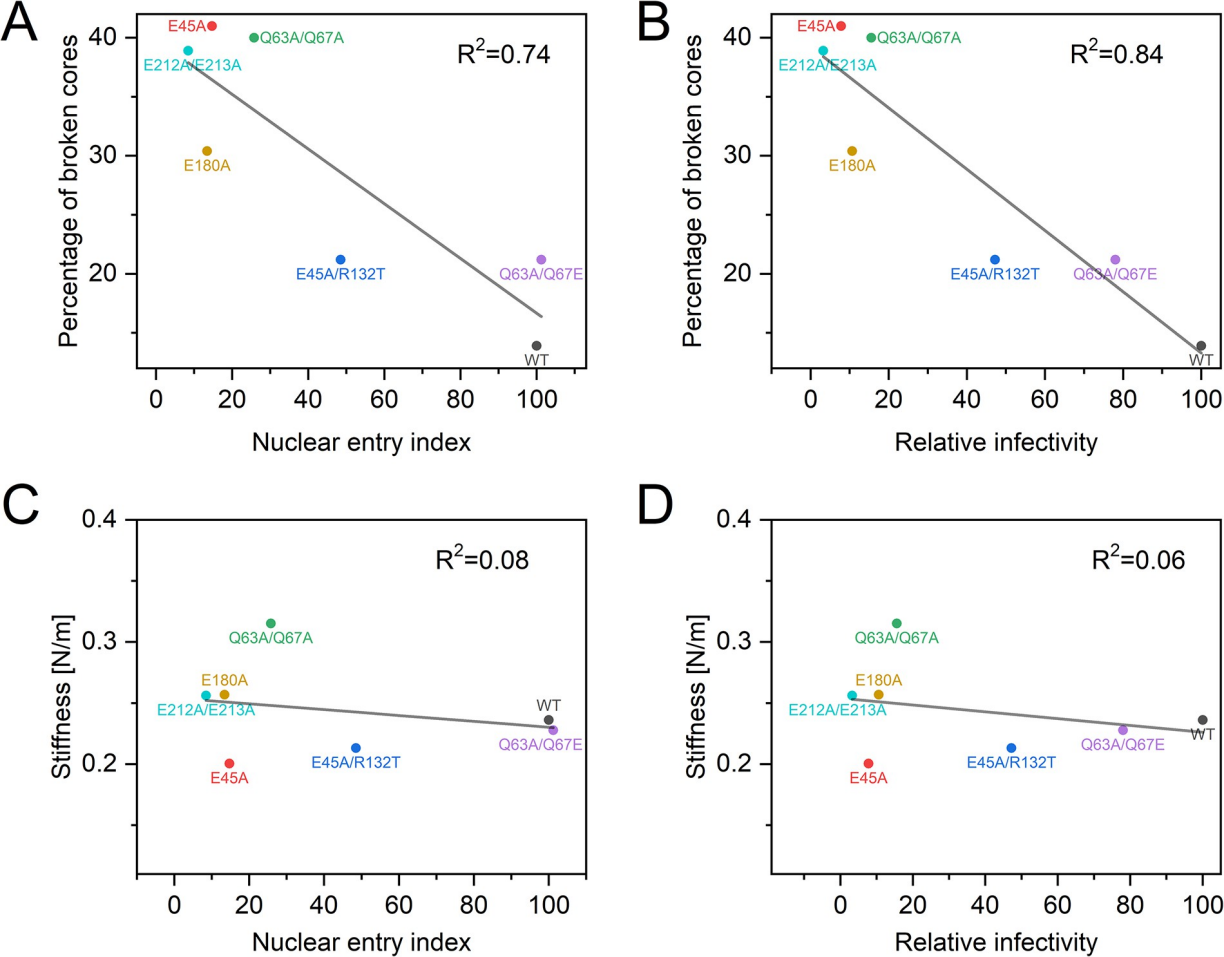
A scatter plot showing an inverse correlation between HIV-1 core elasticity, determined by the presence of broken cores following compression and (A) nuclear entry index and (B) relative infectivity. The core stiffness plotted as a function of (C) nuclear entry index and (D) relative infectivity, revealing no-correlation between these properties. The solid line represents the linear fit of the data.

AFM analysis showed that cores from the E212A/E213A, Q63A/Q67A, and E180A mutant virions were also relatively brittle, exhibiting 40%, 39%, and 32% breakage upon compression, respectively. Substitution of glutamic acid for alanine at position 67 (Q63A/Q67E), a mutation that was acquired during adaptation of the Q63A/Q67A mutant for replication in T cells, restored elasticity to the wild type level (21% of broken cores). A correlation was derived between core elasticity, and nuclear entry, and infectivity (Fig 4A and 4B). To determine whether capsid stiffness may also play a role in HIV-1 nuclear entry, we quantified the stiffness of purified HIV-1 cores using AFM operated in the nano-indentation mode (S9 Fig). We found that the stiffness of the core was not correlated with nuclear entry and infectivity (Fig 4C and 4D).

### Brittle HIV-1 cores are capable of engaging the nuclear membrane, but only elastic cores enter the nucleus

**Fig 5:**
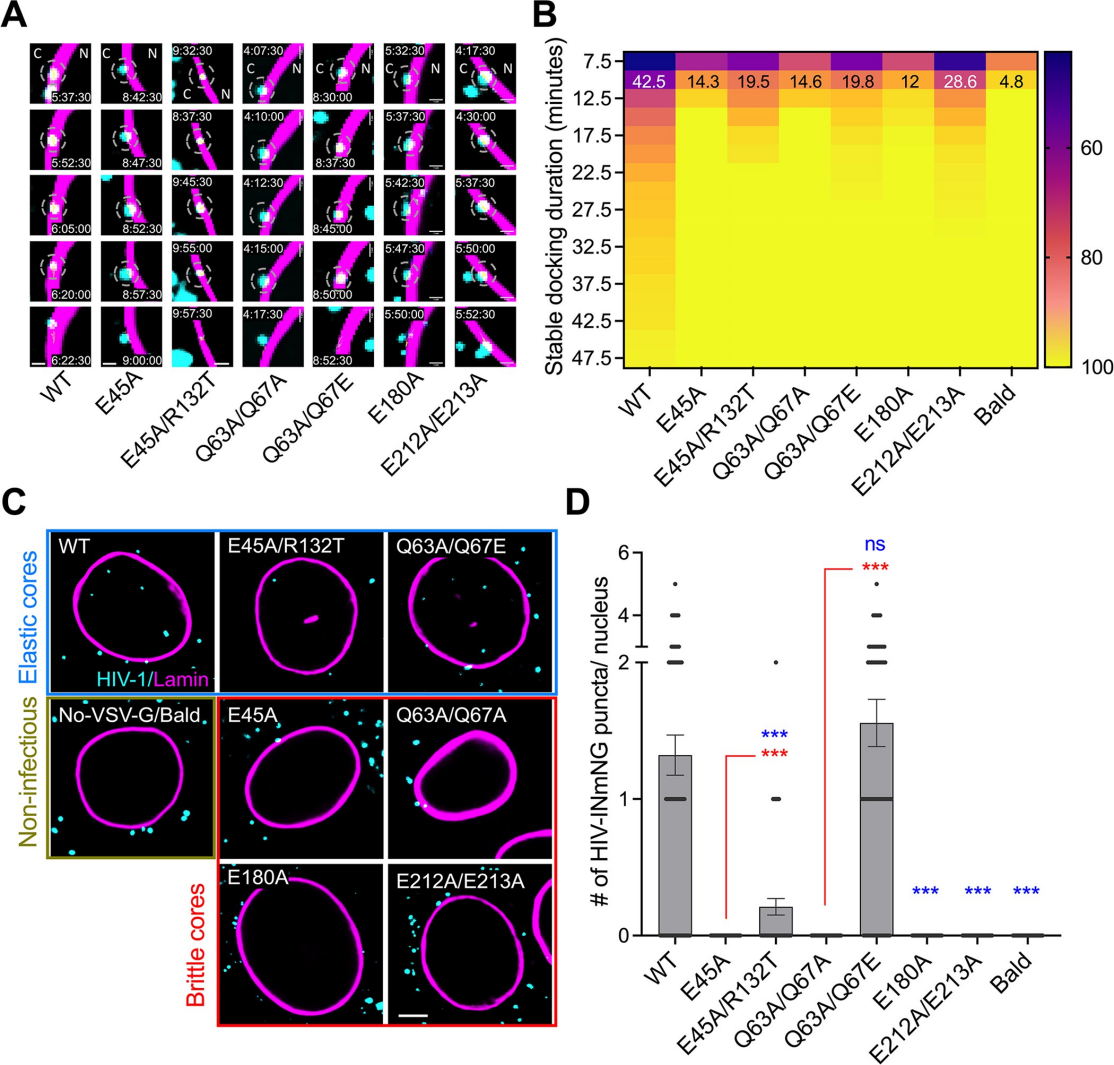
Brittle HIV cores reach the nuclear membrane, but only elastic cores enter the nucleus. (A) Representative time-series images showing interactions between the HIV-1 core (cyan, puncta) and the nuclear lamina (magenta). The segments of stable interaction are marked by dashed circles, time stamps (hh:mm:ss), and location cytoplasm (C) and nucleus (N) are overlayed in images. (B) The cumulative frequency plot shows all stable HIV-1 docking interactions at the nuclear lamina. The fraction of cores interacting for longer than 10 min is overlayed on the heat-map. (C) single z-stack images and (D) quantification of HIV-1 INmNG puncta nuclear entry in TZM-bl cells at 8 hours post-infection. Pairwise student T-test was performed, p-values <0.0001 was considered highly significant ***, and values >0.05 was considered not-significant (ns). Stats in blue is vs. WT and in Red is vs. in-elastic counterparts. Note images in (A), (C) are single z-stacks and quantification in (D) is from the entire nucleus volume from >100 nuclei for independent CA mutants. Scale in (A) is 1 μm, and in (C) is 5 μm.

In principle, mutants with brittle cores may also exhibit impaired nuclear entry owing to premature cytoplasmic capsid breakage, thus failing to traffic to the nuclear membrane. To assess the efficiency at which elastic and brittle cores interact with the nuclear lamina, we employed live-cell fluorescence confocal imaging to visualize over 16h of HIV-1 cores labeled with the Vpr-integrase-mNeonGreen (INmNG) marker in TZM-bl cells (Fig 5) [6,50]. This extended imaging approach allowed us to document interactions between cores and the fluorescently labeled nuclear envelope (NE), including the nuclear entry of INmNG-cores, within the same sample. To facilitate the identification of subtle interactions between cores and the NE, we performed a blinded and unbiased tracking of all single HIV-1 cores that came in proximity of 1-pixel (180 nm) of the NE over an extended 8h time-window (between 4–12 hpi). The resulting single virus trajectories were analyzed in an automated fashion for NE interactions, which was defined as a track containing 3 or more detections on the NE. Next, each individual trajectory was analyzed for segments that showed a docking behavior (stable interactions), which was defined by a highly stringent localized movement of the diffraction-limited HIV-INmNG puncta within a 2-pixel (360 nm) radius on the NE in 3 or more consecutive frames of a track. From this analysis a cumulative frequency plot was derived for all cores that show a docking behavior, and the duration of docking at the NE *vs*. cumulative frequency was analyzed. As a control for the diffraction limited INmNG-puncta tracking along the NE, we analyzed the trajectories of endosomally trapped ‘bald’ HIV-1 cores that were produced without an envelope glycoprotein (no-VSV-G) for its docking behavior, and to estimate the probability of non-specific detection in our method.

Single virus trajectory analysis (S10 Fig) revealed that all mutant cores successfully reached the nucleus (Fig 5A and 5B). Notably, the fraction of cores that exhibited long docking behavior (>10 min,) was >2-fold for the brittle E45A (14.3%), Q63A/Q67A (14.6%), E180A (12%), and E212A/E213A (28.6%) cores, when compared to non-specific endosomal interactions (4.8%) (Fig 5B). This docking frequency was similar for the elastic pseudorevertant mutant cores E45A/R132T (19.5%) and Q63A/Q67E (19.8%), when compared to their inelastic counterparts E45A and Q63A/Q67A, respectively. With the exception of the E212A/E213A, the duration of long-docking behavior for all other mutant cores paled in comparison with the WT cores (42.5%) (Fig 5B).

Whereas, NE docking behavior was preserved for all brittle and elastic cores, albeit to different extents, only the elastic cores (WT, E45A/R132T, and Q63A/Q67E) retained the ability to enter the nucleus. Analysis of over ~100 nuclei in respective individual live cell movies failed to identify the nuclear entry of brittle cores (Fig 5C and 5D). However, the elastic pseudorevertant E45A/R132T cores entered the nucleus at a 5-fold lower frequency, while the Q63A/Q67E mutant entered the nucleus at levels similar to that of the WT HIV-1 core (Fig 5D). The nuclear import frequency of the E45A/R132T and Q63A/Q67E mutants measured by imaging assay here, is consistent with the 2-LTR and infectivity measurements done in independent experiments (Fig 3). Therefore, while all the mutants tested here successfully reached the nuclear envelope, they differ in their ability to traverse the nuclear pore, a property exhibited only by elastic cores.

### PF74 and LCV reduce HIV-1 capsid elasticity

Small molecule inhibitors that target the viral capsid, including PF74 and LCV, are known to inhibit HIV-1 infection by preventing nuclear entry at low concentrations of the compounds [6,17]. These compounds bind to a pocket in the viral capsid that interacts with nucleoporins [17,21,51], suggesting that the inhibitors act by steric inhibition of the capsid binding to the nucleopore. Because the binding pocket resides at the NTC-CTD intersubunit interface, we asked whether these compounds also affect elasticity. Addition of PF74 or LCV to wild type cores rendered them brittle, as reflected by increased frequency of breakage upon compression (39% and 36% for PF74 and LCV, respectively) (Fig 3F). Thus, capsid-targeting small molecules reduce HIV-1 core elasticity at concentrations that inhibit nuclear entry [17]. In a previous study, we showed that E45A and Q63A/Q67A are resistant to PF74, despite retaining the ability to bind to the inhibitor [28]. Infection by the nuclear entry-impaired mutants E180A and E212A/E213A was also resistant to the inhibitors [17]. Both E45A/R132T and Q63A/Q67E were sensitive to the capsid inhibitors (S11 Fig). Therefore, HIV-1 core elasticity is correlated with capsid inhibitor sensitivity and nuclear entry.

**Fig 6:**
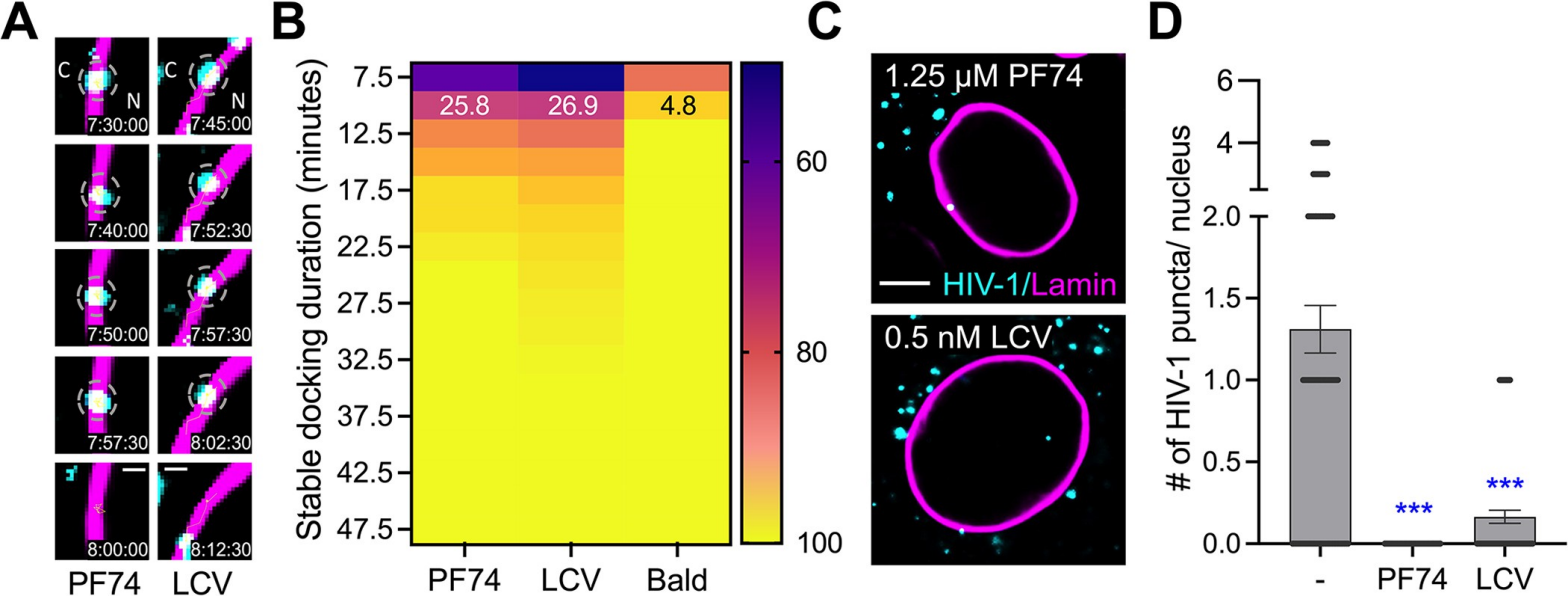
Low concentrations of PF74 and LCV block HIV nuclear import. (A) Representative time-series images showing PF74 (1.25 μM) and LCV (0.5nM) treated HIV-1 core (cyan, puncta) interactions with the nuclear lamina (magenta). The segments of stable interaction (dashed circles), time stamps (hh:mm:ss), and location cytoplasm (C) and nucleus (N) are overlayed in images. (B) The cumulative frequency plot shows all stable HIV-1 docking interactions at the nuclear lamina for PF74 and LCV treatment. The fraction of cores interacting for longer than 10 min is overlayed on the heat-map. For clarity, bald endosomal core interactions (from Fig 5B) is included here. (C) single z-stack images and (D) quantification of HIV-1 INmNG puncta nuclear entry at 8 hours post-infection. Pairwise student T-test was performed, p-values <0.0001 was considered highly significant ***, and values >0.05 was considered not-significant (ns). Note images in (A), (C) are single z-stacks, and quantification in (D) is from the entire nucleus volume from >100 nuclei for independent CA mutants. Scale bar in (A) is 1 μm, and in (C) is 5 μm.

Live-cell single HIV-1 tracking further illustrated that at low-concentration, PF74 and LCV had at best only a modest effect on HIV-1 interaction with the NE (Fig 6A and 6B), but near completely abolished HIV-1 nuclear entry (Fig 6C and 6D). The fraction of capsids showing prolonged interactions with the NE was 25.8% in PF74 and 26.9% in LCV-treated infections. This was a >5-fold increase in the probability of capsids interacting with the NE in a specific manner (compare to bald 4.8% non-specific interactions). Thus, at low concentration of capsid-targeting small molecules, HIV-1 cores can arrive and interact with the NE, but fail to enter the nucleus.

## Discussion

In this study, we found that the HIV-1 core is exceptionally elastic, capable of withstanding loading forces of up to 20 nN without undergoing any detectable damage. More importantly, we demonstrate elasticity as a new property of the HIV-1 core and showed that it is tightly linked to HIV-1 nuclear entry in nondividing cells. In previous studies, we characterized the stiffness of mutant HIV-1 cores and showed that the E45A mutation, which increases capsid stability and stiffness [25,32], can result in impaired infectivity owing to a defect in nuclear entry [26]. A suppressor mutation in CA (R132T) partially rescued infectivity and restored nuclear entry and infectivity, yet the suppressor mutation failed to reverse the hyperstability of the mutant cores, indicating that capsid hyperstability is not the major cause of the nuclear entry impairment associated with the E45A mutant [26]. This conclusion is consistent with recent imaging-based studies indicating that HIV-1 cores enter the nucleus of nondividing cells with little to no disassembly of the capsid [10,12,52]. Furthermore, the nuclear pore itself appears to be of sufficient diameter to accommodate the broad end of the HIV-1 core, suggesting that the pore may not represent a size restriction to HIV-1 nuclear entry [52]. Additionally, the E45A mutation resides at a location in the assembled HIV-1 capsid distant form the nucleoporin binding sites [26,28], further indicating that the mutation alters a properly that is required for nuclear entry and is distinct from nucleoporin binding [27].

In the current study, we observed that the stiffness of the core does not account for the nuclear entry impairment exhibited by the E45A mutant virus. Instead, we linked the nuclear entry defect to a reduction in core elasticity. Using AFM, we observed that wild type HIV-1 cores rapidly returned to their original volume following compression to 30% of their original volume. By contrast, a high fraction of E45A mutant cores underwent breakage in response to compression, indicating a loss of elasticity (or increase in brittleness). We identified three additional HIV-1 capsid mutants exhibiting impaired nuclear entry that also contained cores with decreased elasticity: Q63A/Q67A, E180A, and E212A/E213A. These mutations reside at the three major intersubunit interfaces in the mature HIV-1 capsid, suggesting that capsid elasticity is determined by multiple CA-CA contacts within the lattice. Importantly, cores from the pseudorevertants E45A/R132T and Q63A/Q67E exhibited restored elasticity. By contrast to the parental mutants E45A and Q63A/Q67A, infection by the latter pseudorevertants was resistant to cell cycle arrest by aphidicolin, and the viruses exhibited partial (E45A/R132T) or complete (Q63A/Q67E) restoration of nuclear entry. Thus, HIV-1 nuclear entry was strongly correlated with the elasticity of the viral core, a property that is determined by the viral capsid.

Our computational modeling studies of the HIV-1 capsid also support its elastic property. By modeling the response of the assembled capsid to a force perturbation, we observed that the capsid can be compressed to a certain extent without breaking, and that the E45A substitution in CA reduced this property. Moreover, by simulating the consequences of a high-velocity probe impact at specific locations on the capsid on the integrity of the viral capsid, we observed capsid failure at shorter indentation distances for the E45A mutant vs. wild type, suggestive of a decrease in its ability to withstand a strongly applied force without breaking. By contrast, the response of the E45A/R132T mutant capsid was like that of the wild-type capsid, consistent with a restoration of capsid elasticity as observed in our AFM experiments.

Using live-cell imaging we show that all the brittle cores can arrive and interact with the nuclear envelope. The duration of interactions with the NE was similar for the brittle (E45A, and Q63A/Q67A) and their respective pseudo-revertants (E45A/R132T and Q63A/Q67E), suggesting that their inability to enter the nucleus was not limited by their ability to arrive at or interact with the nuclear pore. It is additionally possible that inelastic cores could undergo breakage due to their brittleness, leading to their reduced engagement with the nuclear pore complex that we observed (Fig 6B). These could perhaps occur during reverse transcription in the cytoplasm, which may additionally account for activation of the cytoplasmic DNA sensor cGAS, as reported recently [53]. Additional studies will be required to determine the extent to which this occurs with the mutants.

HIV-1 capsid-targeting small molecule inhibitors, PF74 and LCV occupy a pocket in the assembled capsid in which nucleoporins also bind, suggesting steric interference of nucleoporin engagement as a mechanism of inhibition of nuclear entry [17,28,48]. We observed that low concentrations of these inhibitors had only modest effect on the ability of HIV-1 cores to interact with the nuclear membrane, however, they selectively reduced the elasticity of the viral core and blocked nuclear entry. It is also feasible that the docking efficiency measured in this work (Figs 5 and 6) reflects the possibility of HIV-1 cores interacting with the Cyp-domain of the nucleoporin NUP358 present in the cytoplasmic side of the NPC. In the present study, we showed that four HIV-1 CA mutants exhibiting resistance to PF74 and LCV contain cores exhibiting reduced elasticity vs. the wild type. Two pseudorevertants, E45A/R132T and Q63A/Q67E, exhibited cores with restored elasticity and increased sensitivity to the inhibitors. We conclude that HIV-1 core elasticity is important for nuclear entry and for sensitivity to capsid inhibitors that prevent it.

Our finding that elasticity is important for nuclear entry and that the inhibitors reduce core elasticity suggests a dual mechanism of inhibition of HIV-1 nuclear entry. The resistance of the inelastic capsid mutants to the inhibitors is also consistent with the inability of the mutants to utilize the nuclear pore complex for nuclear entry. Accordingly, the pseudorevertants E45A/R132T and Q63A/Q67E were more sensitive to inhibition by PF74 and LCV, likely owing to their restored ability to enter the nucleus via the nuclear pore complex.

While our study supports a requirement for core elasticity in HIV-1 nuclear entry, the basis for this requirement remains to be determined. A current view is that the nuclear pore is large enough to permit passage of the intact HIV-1 core [52], yet the pore forms a size-selective barrier for passage of cargo, with large cargoes requiring shuttling by transporter proteins [54]. The pore is normally occupied by a dense array of nucleoporin side chains forming a gel-like barrier through which cargo must pass [55–57]. There is compelling evidence that the HIV-1 capsid interacts directly with nucleoporins [49], and it seems plausible that elasticity is required for accommodating the potential distortion that may be exerted on the core during multivalent interactions with central nucleoporin side chains in the channel. Currently, the molecular events leading to traversal of a docked HIV-1 core through the nuclear pore are undefined, though interactions with nucleoporin FG sequences are likely involved. While the reduction of apparent residence time at the nuclear membrane by nuclear entry-defective mutants could result from expulsion following docking, the elasticity of the core may also be important for the capsid to accommodate the internal structural changes that likely occur during passage through the central channel of the nuclear pore.

Among retroviruses, lentiviruses such as HIV-1 are unusual in two properties: the conical shape of their cores and the ability to infect nondividing cells, both of which are determined by the capsid. Purified HIV-1 CA protein can self-assemble into cones in vitro [32,58], and it is possible that the conical shape is responsible for the efficient ability of HIV-1 to pass through the nuclear pore complex. In addition to nucleoporin binding, our results suggest that elasticity is a key property of the HIV-1 core that facilitates its passage through the nuclear pore complex. Whether elasticity is a property intrinsic to conical capsids remains unclear, future work will be needed to address this question.

## Materials and methods

### HIV-1 core production and core purification

Human embryonic kidney (HEK) 293T cells were used for the preparation of HIV-1 pseudovirus particles as previously described [43]. Briefly, ~10^6^ HEK 293T cells were cultured in a 15 cm dishes and transfected with 2.5 μg of Env-defective variants of the full-length HIV-1 construct R9 using 10 μg of polyethyleneimine (PEI; branched; MW ~25000; Sigma-Aldrich). After 20 h, the cell medium (Dulbecco’s modified Eagle medium, supplemented with 10% heat-inactivated fetal bovine serum, 1% penicillin-streptomycin, and 1% L-glutamine) was replaced with fresh medium. After 6–7 h, the virus-containing supernatant was harvested, centrifuged at 1,000 rpm for 10 min, and filtered through a 0.45 μm pore size filter. The supernatant was concentrated using ultracentrifugation in an SW-28 rotor (25,000 rpm, 2 h at 4°C) on a 60% OptiPrep density cushion (Sigma-Aldrich). The pelleted viruses were resuspended in 10 mL TNE buffer (50 mM Tris-HCl, 100 mM NaCl, 0.1 mM EDTA; pH 7.4), and added to 100-kDa molecular mass cutoff Vivaspin 20 centrifugal concentrators (100,000 MWCO; Sartorius AG, Germany). The mixture was centrifuged twice at 2,500 ×g for 25–30 min at 4°C until the supernatant level in the concentrator reached a final volume of 300–350 μL.

Virus cores were isolated from the concentrated virus-containing supernatant using a previously described protocol [43]. Purified HIV-1 virus particles were mixed with 1% Triton-X 100 diluted in 100 mM of 3-(N-morpholino) propane sulfonic acid (MOPS) buffer (pH 7.0) and incubated on ice for 2 min. The mixture was centrifuged at 13,800 g for 8 min at 4°C. After removing the supernatant, the pellet was washed twice using ~80 μL of MOPS buffer and repelleted. The final pellet was resuspended in a 10 μL MOPS buffer. A fresh batch of cores was prepared on the same day as each of the AFM measurements. When specified, IP6, PF74, or Lenacapavir were added to the buffer at a final concentration of 100 μM, 1.25 μM, or 500 pM, respectively.

### AFM measurements and analysis

Samples for AFM measurements and analysis were prepared as previously described [43]. Briefly, 10 μL of isolated HIV-1 cores were incubated for 45 min at room temperature on hexamethyldisilazane (HMDS)-coated microscope glass slides (Sigma-Aldrich) in a mildly humid chamber. AFM measurements were performed on the adhered sample without fixation. Each experiment was repeated at least three times, each time with independently purified pseudoviruses. IP6 was received as a gift from Leo James. PF-3450074 and Lenacapavir (GS-6207, Gilead) were purchased from MedChemExpress (MCE), USA. All measurements were carried out with a JPK Nanowizard Ultra-Speed atomic force microscope (JPK Instruments, Berlin, Germany) mounted on an inverted optical microscope (Axio Observer; Carl Zeiss, Heidelberg, Germany). Silicon nitride probes (mean cantilever spring constant of 0.12 N/m; DNP, Bruker, Germany) were used. Topographic images were acquired using the quantitative imaging (QI) mode at a rate of 0.5 lines/s and a loading force of 300 pN. Briefly, QI mode is a force-curve based imaging mode in which the height at each pixel is determined by the movement of the Z-piezo from a fixed height until the determined maximal force is achieved. All images were rendered using the WSxM software (Nanotec Electronica).

#### Stiffness measurements

Core stiffness was obtained by operating the AFM in nanoindentation mode as previously described [32,43]. Briefly, to determine the stiffness of each core, 20 force-distance (FD) curves were obtained from 24 different points on the core surface for a total of 480 FD-curves per core. To confirm that the capsid remained stable during the entire indentation experiment, each experiment was analyzed by the plotting individual point stiffness as a histogram and as a function of the measurement count. Samples whose point stiffness values decreased consistently during measurement, indicative of irreversible deformation, were discarded. The number of discarded FD-curves was 1 from each sample (corresponding to ~3.5%). Importantly, this small number of discarded cores was similar for all samples, regardless of their elasticity. The maximal indentation of the sample surface was 4 nm which corresponds to a maximum loading force of 0.2–1.5 nN. Each FD-curve was shifted to set the deflection in the non-contact section to zero (baseline). The set of FD-curves was then averaged. The measured stiffness was mathematically derived from the slope of the FD-curves. Core stiffness was computed using Hooke’s law, assuming that the experimental system can be modeled as two springs (the core and the cantilever) arranged in series. The spring constant of the cantilever was determined during experiments by measuring the thermal fluctuations. The data were analyzed using JPK Data Processing and MATLAB (The Math Works, Natick, MA) software. To test the mechanical strength of the core, a single FD curve was acquired from a single central point on the surface of the core. The maximum loading force was set to either 5 nN, 10 nN or 20 nN.

#### Volume recovery measurements

The elasticity of the core was assessed in terms of volume recovery after forcibly compressing the structure. The entire core was imaged using the AFM operated in the QI mode using a low maximal force of 300 pN. A small rectangular region on the main body of the core was then selected and scanned. The chosen region was then compressed by re-scanning it at a maximal loading force of 5 nN. Higher maximal loading forces frequently resulted in the detachment of cores from the glass substrate as evident by the disappearance of the core in the consequent images. The force was then lowered back to 300 pN, and the region was repetitively imaged for a total duration of 5–6 minutes. At the end of the experiment, the entire core was imaged at a 300 pN loading force. The collected set of images was then processed using an in-house MatLab script to calculate the volume underneath the selected region. The initial volume prior to compression was set to 100%, and the rest of the calculated volumes were normalized accordingly. The obtained volume values were then plotted against time. The initial and final images of the entire core were used to determine if the core underwent structural breakage upon compression. Breakage was assessed as the loss of a significant part of the core when the before and after compression images are compared.

### HIV-1 infection assays

Infectivity was quantified by titration of GFP reporter viruses bearing the indicated amino acid substitutions in CA. Virus stocks were prepared by cotransfection of 293T cells with 3 μg of Env-defective HIV-GFP proviral plasmid [59] and 0.5 μg pHCMV-G [60], generating VSV-pseudotyped particles. After culturing for 40–48 h, virus-containing culture supernatants were collected, clarified by passing through 0.45 μm syringe filters, frozen in aliquots, and stored at -80°C. Virus concentrations were determined by p24 ELISA [61]. Infections were performed by inoculating 20,000 Hela cells seeded one day previously in 48-well plates with 0.25 ml of virus dilutions prepared in complete medium containing 20 μg/ml DEAE-dextran. Cultured were incubated for two days and cells detached by treatment with trypsin, collected, fixed overnight in 0.4% paraformaldehyde, and analyzed for GFP expression by flow cytometry. Infectivity was determined as % GFP^+^ cells in the culture normalized by the concentration of p24 in the inoculum with wells exhibiting between 1% and 20% GFP^+^ cells. To assay inhibition of infection by PF74 and LCV, the compounds were added to a dilution of each virus that gave 5–10% infected cells in the absence of inhibitor. Two days later, the cells were fixed and analyzed for infection by flow cytometry.

In experiments involving cell cycle arrest, Hela cells (40,000) were seeded in medium containing aphidicolin (1 μg/ml). The next day, medium was removed, and aphidicolin was included in the virus inocula. The following day, the medium was replaced with fresh medium lacking aphidicolin. In experiments involving Nup153 depletion, cells were first transfected with control or Nup153-targeting siRNA duplexes as previously described [48]. Two days later, cells were replated for infection assays and for immunoblotting to confirm the knockdown efficiency.

To isolate the Q63A/Q67E pseudorevertant, cultures of MT-4 cells (200,000) in 1 ml of medium were inoculated with R9.Q63A/Q67A mutant virus (25 ng p24) encoding a Vpu truncation at amino acid 35. The cultures were maintained and assayed for HIV-1 accumulation by p24 ELISA every four days. After approximately two weeks, p24 was detected in the mutant virus culture. DNA was extracted and purified using a DNAeasy kit (Qiagen), and the product used as a template for PCR amplification of the Gag coding region. PCR products were gel purified, sequenced, and cloned into the wild type R9 and HIV-GFP reporter virus plasmids. The PCR-amplified regions of the plasmids were sequenced to confirm identity.

For quantifying reverse transcription and nuclear entry, monolayers of 200,000 Hela cells in 12-well dishes were inoculated with 0.5 ml of virus, corresponding to 20 ng of p24, that had been previously treated with DNaseI to reduce the level of contaminating plasmid DNA. Infection was performed in medium containing 20 μg/ml DEAE-dextran. When required, Efavirenz or Raltegravir was added to diluted viruses to a final concentration of 1 μM. Cultures were incubated for 16 hours, cells were collected, and DNA was purified on silica columns [62]. DNA was eluted in 100 μl of water, quantified by spectrophotometry, and assayed for late reverse transcripts and 2-LTR circles by quantitative PCR with standard curves generated from dilutions of appropriate plasmids.

### Simulated AFM nanoindentation experiments

#### HIV-1 CA conical capsid construction

The coarse grained HIV-1 conical capsid was modeled using a methodology we developed previously[63]. Details of the coarse graining methodology, as well as parameterization and model validation, were previously reported [63]. The system includes models of sodium, chloride, and host-factor inositol hexakisphosphate (IP6), yielding 150 mM NaCl ionic strength and 253 IP6 molecules bound to central pores of capsomers [64]. The charge-neutral, ionized and IP6-bound capsid was then equilibrated for 500 ns using NAMD3 [65].

#### Molecular dynamics simulations

All molecular dynamics (MD) simulations were performed with the nanoscale molecular dynamics (NAMD) engine [65]. NAMD 2.14 was utilized for energy minimization and the fully GPU-resident NAMD 3 was utilized for NVT equilibration and constant velocity steered MD (cv-SMD) simulations. Simulations of wild type capsids utilized a time step of 48 femtoseconds, whereas mutant E45A and E45A/R132T capsid simulations utilized a time step of 40 femtoseconds. Long range electrostatic evaluation was accomplished via particle mesh Ewald (PME) [65,66]. The non-bonded potential excluded bonded neighbors (1–2 exclusion policy) and utilized a cutoff of 2 nm. Short range non-bonded interactions were computed at every time step, while long range interactions were computed every other time step. The discrete grid used in PME for interpolating charges was given a spacing of 2 Å, employing 8^th^ order interpolation. Temperature was enforced via Langevin dynamics, with a target temperature of 298 K. All beads besides the AFM tip were coupled to the thermal bath with a γ coefficient of 2 ps−1. A dielectric constant of 80 was employed to mimic charge screening by solvent molecules [67].

#### In silico AFM simulations

For AFM simulations, a constant velocity pulling was applied to a dummy atom with a velocity of 1.5 × 10^−6^ Å per time step, or 3.125 nm per microsecond. A harmonic force constant of 12 kcal/mol was employed to restrain the 13 nm AFM tip’s center of mass to the dummy atom. Additionally, a transverse harmonic force constant of 1 kcal/mol was employed. Data from the AFM simulations, namely forces acting on the AFM tip throughout simulation as well as the tip’s position data, were output with a frequency of 48 picoseconds, or every 100 time steps (A detailed description of the AFM simulations is provided in the S1 Text).

### Fluorescently virus production

Fluorescently tagged VSV-G pseudotyped HIV-1 particles was produced as described previously [6]. Briefly, envelope deleted pHIVeGFP CA wild-type (WT) and indicated CA mutants (MT) proviral backbone (2ug), VSV-G envelope (0.5μg) and Vpr-integrase fused to mNeonGreen (INmNG, 0.8μg) was mixed in jetPRIME buffer and 6ul of jetPRIME reagent, and transfected into HEK 293T cells plated at 80% confluency in a 6-well plate. Following a 6h incubation in a CO_2_ incubator, the transfection medium was exchanged for fresh phenol-red minus DMEM complete with antibiotics and 10% FBS. Virus supernatants were collected after an additional 36h of incubation, clarified through a 0.45 μm filter and quantified for RT-activity, aliquoted and stored at -80°C until use.

### Live-cell imaging of HIV-1 docking at the nuclear envelope and nuclear import

Single HIV-1 infection in live cells was visualized as previously described [6,68]. In brief, 5 × 10^5^ TZM-bl cells that stably express the emiRFP670-laminB1 nuclear envelope marker was plated in a 35mm 1.5 glass bottom dish (#D35-20-1.5-N, CellVis). Aphidicolin (Sigma Aldrich) was added to cells (10 μM final concentration) to block cell division. About 14h later the cells were infected (MOI 0.2) with indicated WT and mutant HIVeGFP particles fluorescently tagged with Vpr-integrase-mNeongreen (INmNG) to label the viral core. Virus binding to cells was augmented by spinoculation (1500×g for 30 min, 16°C), and virus entry was synchronously initiated by adding pre-warmed complete DMEM medium containing Aphidicolin (10 μM) to samples mounted on a temperature- and CO_2_-controlled microscope stage. 3D time-lapse live cell imaging was carried out on a Leica SP8 LSCM, using a C-Apo 63x/1.4NA oil-immersion objective. Tile-scanning was employed to image multiple (4x4) fields of view. Live-cell imaging was performed starting from 0.5–16 hpi by acquiring 9–11 Z-stacks spaced by 0.8 μm every 2.5 min. The Adaptive Focus control (AFC, Leica) was utilized to correct for axial drift. 512 x 512 images were collected at 180 nm pixel sizes at a scan speed of 1.54 μs pixel dwell time. Highly attenuated 488 and 633 nm laser lines was used to excite the INmNG and emiRFP670-Lamin B1 fluorescent markers and their respective emission was collected between 502–560 nm and 645–700 nm using GaSP-HyD detectors. 3D-image series were processed off-line using ICY image analysis software (http://icy.bioimageanalysis.org/) [69]. More details are provided in the S2 Text.

### Image analyses

HIV-1 nuclear import was assessed by extracting the 3D z-stack images at 8h time-point in the live-cell movies and the images were analyzed using an in-house script in the ICY protocols’ module as described in [68]. For analysis of HIV-1 docking at the nuclear envelope, we selected >10 cells from each independent live-cell experiments that showed minimal lateral movements. The initial- (0-4h) when there is a higher density of INmNG puncta in the cytoplasm and latter (12-16h) segments showing extensive cellular motion in the live-cell movies was removed from the analysis. The central 8h segment (between 4-12hpi) was chosen to facilitate robust and high confidence particle tracking. Analysis was further delimited to the central z-planes (2x planes, 1.6 μm) of these nuclei, which were extracted and projected to a 2D-stack. Images were additionally drift corrected using Fast4DReg in Fiji [70] and were denoised using N2V [71]. The laminB1 fluorescence signals in the processed images was used to mask the pixels occupied by the NE to create a region of interest (ROI). The HIV-1 INmNG puncta detected within this NE mask was tracked in an automated fashion in ICY bio-image analysis single particle tracking suite. Tracks that correspond to 3 or more frames (>7.5 min) were considered as NE-interactions. Each time point in a track was used as a center to determine the number of consecutive frames that a single HIV-1 particle remained localized within a 2-pixel (360 nm) radius for 3 or more frames. The duration of such constricted motion was deemed as stable interactions (docking) with the NE. The 360nm radius was used taking into consideration the lateral diffraction limited confocal resolution (~240 nm) and the pixel sizes (180 nm) in our time-lapse movies. The cumulative frequency of all stable interactions obtained from >10 individual nuclei over the 8h time-window was analyzed and plotted. As controls, the cumulative frequency of background non-specific overlaps of bald endosome trapped HIV-1 (no-VSV) cores at the NE lasting for longer than 10 min was estimated to be 4.8%.

## Supporting information

S1 Data Raw data. (XLSX)

S1 Fig Topographic AFM images of isolated IP6-treated WT cores before and after FD curves applied at maximal loading force of 5 nN, 10 nN or 20 nN. Three representative pairs of images for each maximal loading force. All images were acquired using the QI mode at a maximal loading force of 300 pN. Scale bars are 60 nm.
(TIF)

S2 Fig Simulated AFM volume recovery experiment of a wild type capsid. Following a rapid indentation, the capsid is shown to recover its volume over a relatively short interval of 20 ns. This full and rapid recovery of capsid volume is consistent with experimental volume measurements shown in Fig 4C.
(TIF)

S3 Fig Topographic AFM images of isolated IP6-treated cores. All images were acquired using the QI mode at a maximal loading force of 300 pN. The region that was compressed at 5 nN loading force is shown in a yellow dashed rectangular. Selected cores are shown from all cores analyzed (WT and mutants) and are group based on whether they broke or not following compression.
(TIF)

S4 Fig Simulated atomic force microscopy indentations (3.125 nm/microsecond probe velocity) for **A** wild type, **B** E45A, and **C** E45A/R132T capsids. Each row of the composition represents a different probe location corresponding to those shown in **Fig 1B–1E** and are labeled accordingly. For each plot, probe approach and initial adhesion to the sample surface were omitted, such that the x-axis shows sample indentation in units of nanometers. The linear fits of the first four nm of indentation, from which stiffness values were computed, are shown with the relevant stiffness value annotated. E45A and compensatory E45A/R132T mutant capsids are considerably stiffer than wild type in all locations probed. Wild type stiffness: 0.077 +/- 0.045 N/m; E45A stiffness: 0.309 +/- 0.099 N/m; E45A/R132T stiffness: 0.225 +/- 0.078 N/m.
(TIF)

S5 Fig Summary of high-velocity AFM simulation measurements to evaluate the material response of the HIV-1 capsid in the high-force regime. Shown are the values of (A) power, (B) indentation, and (C) force at which the capsid is ruptured. Mean values from n = 5 simulations are shown, with error bars representing the standard deviations. For panels B and C, position #4 values are omitted; failure of the capsid lattice, and thus critical indentation and force values, is not well-defined due to the relatively small height of the sample in the narrow end.
(TIF)

S6 Fig One of five exemplary trials of high velocity (312.5 nm/microsecond) atomic force microscopy simulations for three simulated capsids: wild type, E45A, and E45A/R132T. Utilizing a higher probe velocity mimics high-force AFM experiments, where we observed deformation and failure of capsids conferring higher measured forces. In the case of simulations, these events were observed across smaller time intervals. Each column is labeled according to the relevant construct, and each row represents a different probe location along the length of the cone (corresponding to **Fig 1B–1E**). Each plot is annotated with the power, determined by integration (shown in green), that is summarized in S8 Fig. Integration bounds are shown, the lower of which was set at probe contact and the upper bound is set to encapsulate the yield (critical) force, annotated with a red dot. Power is considered as the time-normalized integral of each curve (green region), presented in units of picowatts with absolute integration error given. For the narrow end probe locations, position four, critical forces and indentation distances are ill-defined due to densification of the capsids. For the latter cases, we set the upper integration bound as immediately prior to densification. This enables the calculation of power but not critical forces or critical indentation values as shown in S4 Fig for the other three probe positions.
(TIF)

S7 Fig Visualization of deformed and ruptured capsids from AFM simulations. Pre- and post-failure snapshots are shown for: (A) wild type, (B) E45A, and (C) E45A/R132T capsids, with each probe position shown. The velocity of the probe was 312.5 nm/microsecond. Panels **D**-**F** show a succession of close-up images of wild type capsid indentation and failure, including a complete view as well as a C-terminal domain only view, to highlight the separation of assembly interfaces. **E** shows indentation without significant separation of assembly interfaces, slight separation of trimer interfaces is visible. **F** shows the failure event fully manifest, where a large discontinuity is seen in both the complete and C-terminal domain views. It is worth noting that, as opposed to experimental breakages observed, the features of these lattice failures are highly localized. The latter is owed to the rapid probe velocity employed in simulations, and possibly the geometry employed. S13 Fig shows a view of the undeformed capsid employed in each simulation.
(TIF)

S8 Fig Stiffness values from simulated AFM nanoindentation high velocity (312.5 nm/microsecond) FD curves (as shown in S6 Fig), where bars represent the mean of n = 5 trials for each probe position, and for each construct: wild type, E45A and E45A/R132T, colored accordingly. Error bars represent the standard deviation of these five trials. Interestingly, using a significance threshold of 0.05, we see that wild type is significantly stiffer than either mutant at broad end position #1 (p-value = 0.02358 for E45A vs. wild type; p-value = 0.01089 for E45A/R132T vs. wild type). For narrow end position #4, E45A is significantly stiffer than wild type (p-value = 0.006611). For the remaining probe positions, all three constructs confer insignificant differences in stiffness, with p-values > 0.05. This conforms to experimentally derived stiffness trends of wild type, E45A and E45A/R132T capsids.
(TIF)

S9 Fig Averaged measured point stiffness of isolated HIV-1 cores. Each stiffness value was calculated as the average of ~480 force–distance curves obtained from individual cores. Measurements were conducted in the presence of inositol hexakisphosphate (IP6; 100 μM). The t-test analysis revealed that differences between the stiffness values of the various samples and WT are statistically significant (p values: <0.001 and <0.05 for WT+IP6+PF74 and E45A+IP6, respectively, <0.0001 for the remaining samples). Error bars represent the standard error of the mean.
(TIF)

S10 Fig Single HIV-1 docking analysis for WT and capsid mutants interacting with the nuclear envelope. Docking segments (green) and displacements (red) are highlighted. Scale bar is 0.36 μm.
(TIF)

S11 Fig Effects of 0.25 nM Lenacapavir (LCV) and 1.25 μM PF74 on infection by HIV-1 CA mutants. Error bars represent standard deviation of the mean.
(TIF)

S12 Fig Topographic AFM images of broken IP6-treated cores before and following high-force (5 nN) compression. Two representative images for each mutant and WT treated with PF74 or LCV are shown. All images were acquired using the QI mode at a maximal loading force of 300 pN. Scale bars are 60 nm.
(TIF)

S13 Fig View of the undeformed HIV-1 capsid employed for simulated nanoindentations. The capsid is shown whole (left) and clipped (right). For the N-terminal domain, capsid hexamers are colored tan and capsid pentamers are colored green. C-terminal domains are colored blue. Orange beads in the centers of each capsomer represent IP6.
(TIF)

S1 Text A detailed description of the AFM simulations. (DOCX)

S2 Text Additional information for live-cell imaging of HIV-1 docking a the nuclear envelope and nuclear import. (DOCX)
