# Supplementary figures and images for: Elasticity of the HIV-1 core facilitates nuclear entry and infection
Source: PLoS Pathog. 2024 Sep 11;20(9):e1012537. doi: 10.1371/journal.ppat.1012537 (PMC11419384; doi:10.1371/journal.ppat.1012537)

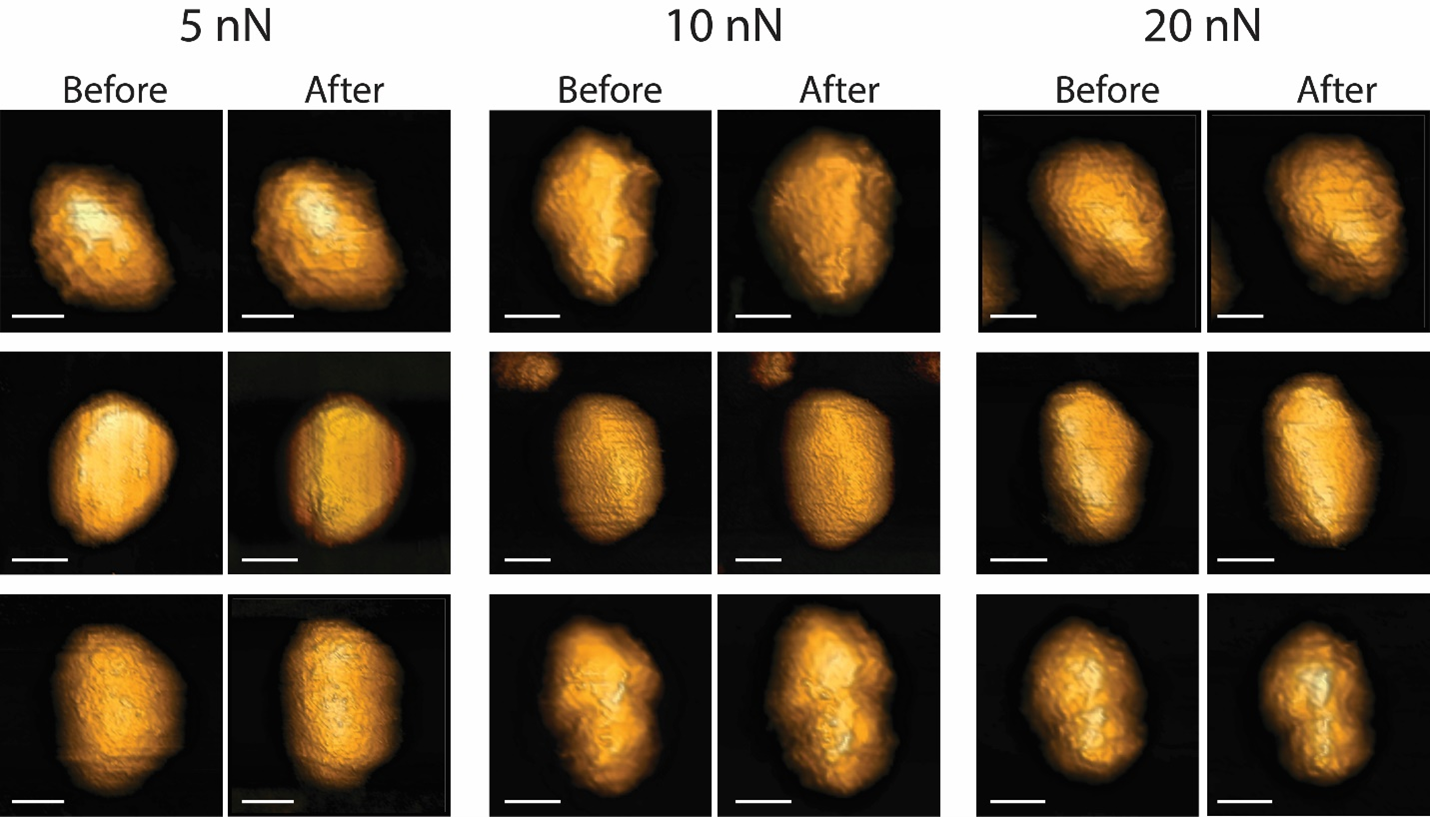

Supplement: S1 Fig — Three representative pairs of images for each maximal loading force. All images were acquired using the QI mode at a maximal loading force of 300 pN. Scale bars are 60 nm. (TIF) [file ppat.1012537.s002.tif]

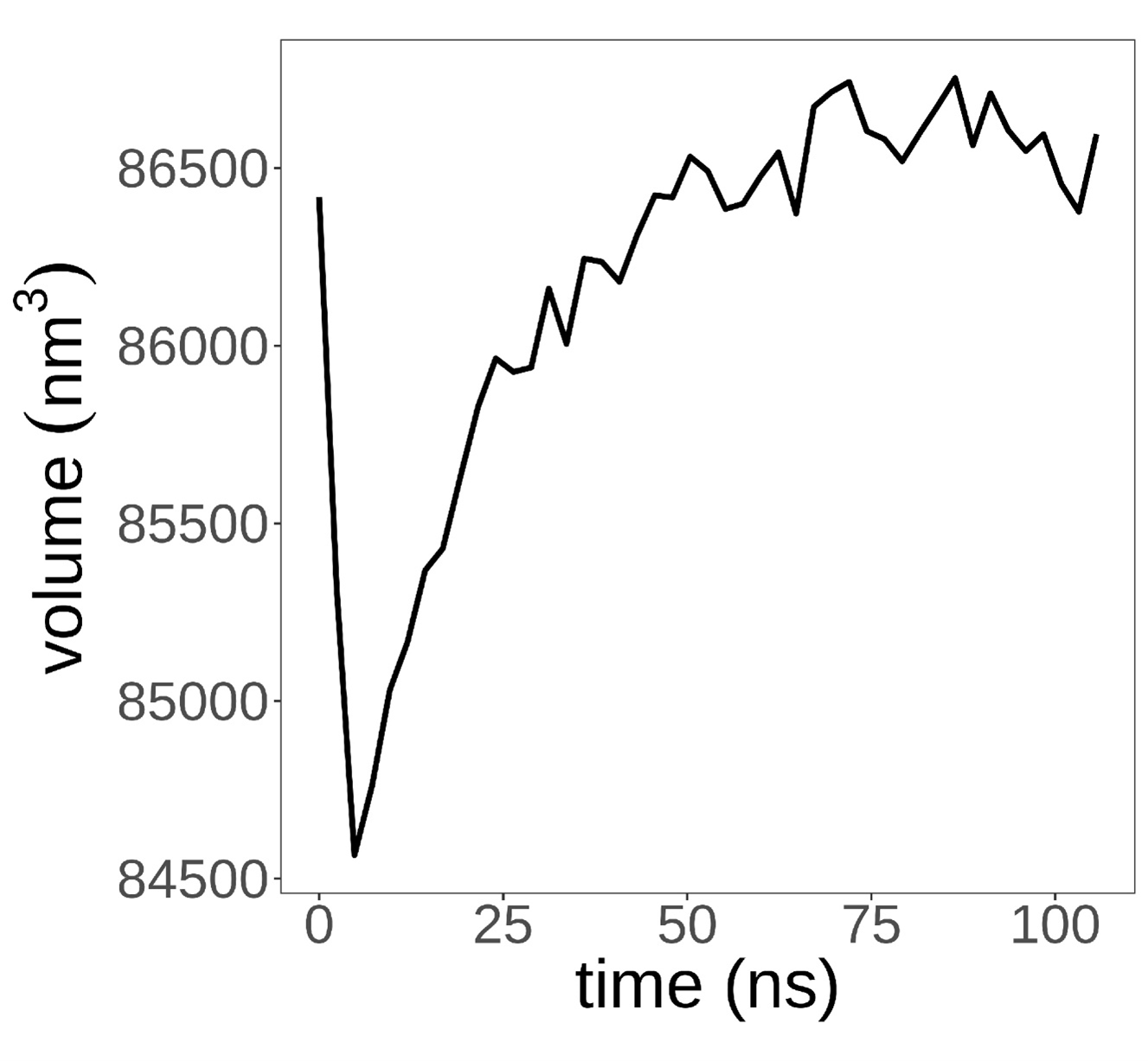

Supplement: S2 Fig — Following a rapid indentation, the capsid is shown to recover its volume over a relatively short interval of 20 ns. This full and rapid recovery of capsid volume is consistent with experimental volume measurements shown in Fig 4C. (TIF) [file ppat.1012537.s003.tif]

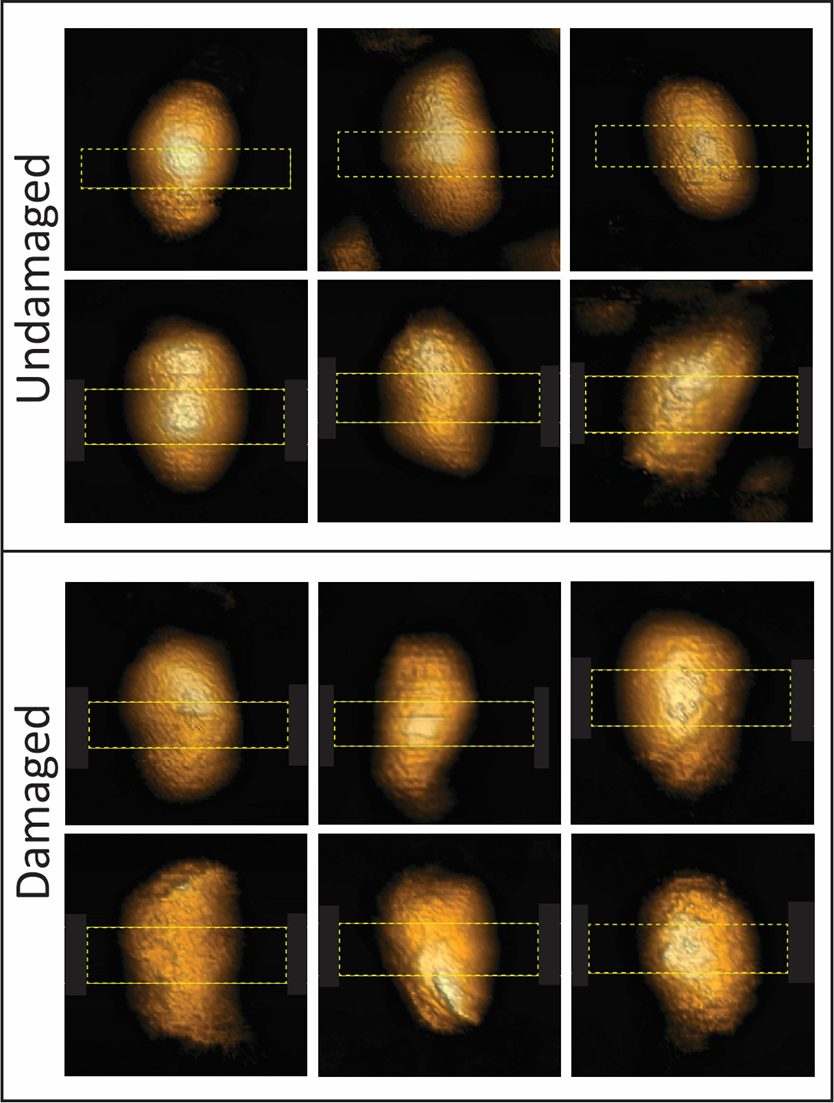

Supplement: S3 Fig — All images were acquired using the QI mode at a maximal loading force of 300 pN. The region that was compressed at 5 nN loading force is shown in a yellow dashed rectangular. Selected cores are shown from all cores analyzed (WT and mutants) and are group based on whether they broke or not following compression. (TIF) [file ppat.1012537.s004.tif]

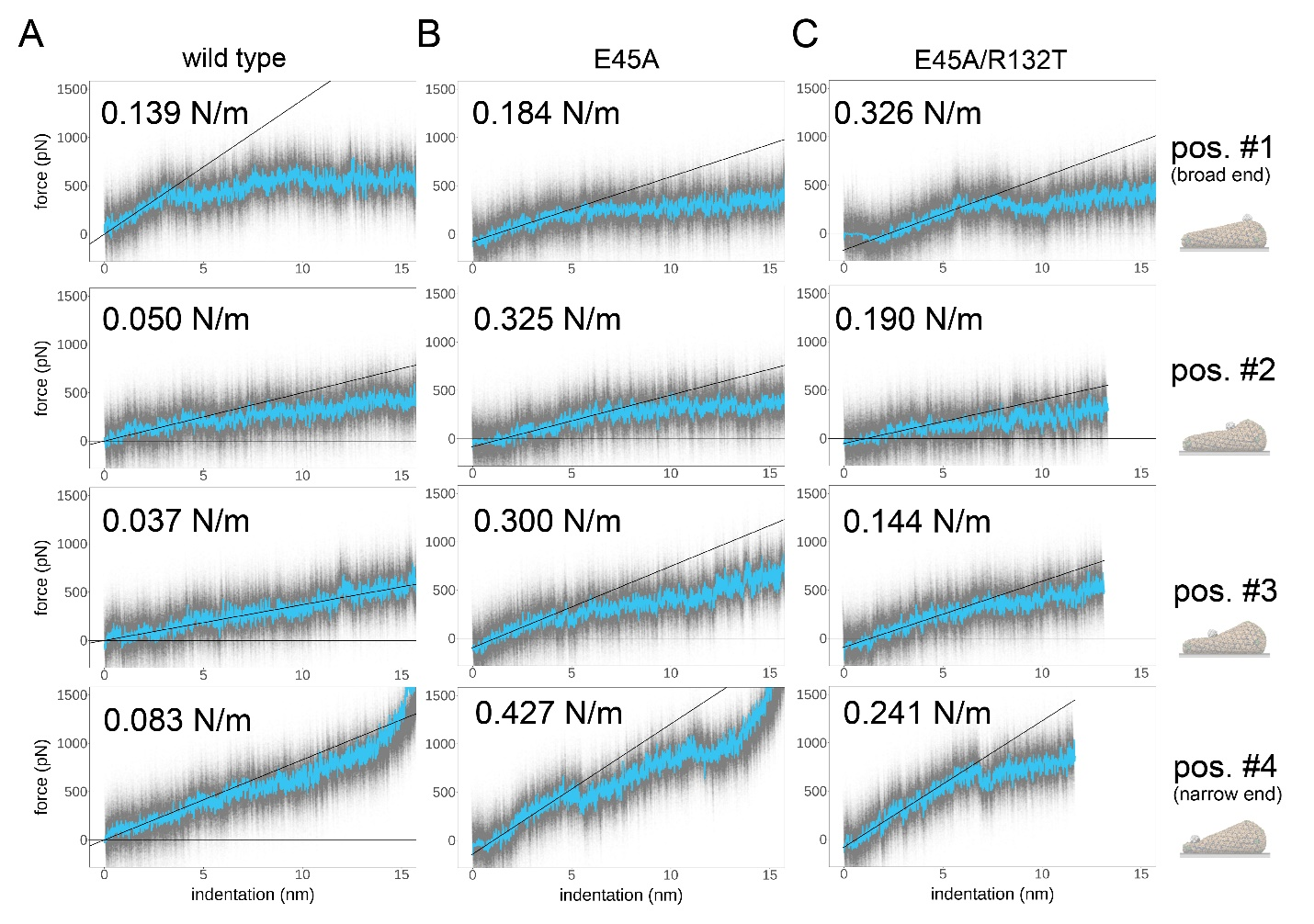

Supplement: S4 Fig — Simulated atomic force microscopy indentations (3.125 nm/microsecond probe velocity) for A wild type, B E45A, and C E45A/R132T capsids. Each row of the composition represents a different probe location corresponding to those shown in Fig 1B–1E and are labeled accordingly. For each plot, probe approach and initial adhesion to the sample surface were omitted, such that the x-axis shows sample indentation in units of nanometers. The linear fits of the first four nm of indentation, from which stiffness values were computed, are shown with the relevant stiffness value annotated. E45A and compensatory E45A/R132T mutant capsids are considerably stiffer than wild type in all locations probed. Wild type stiffness: 0.077 +/- 0.045 N/m; E45A stiffness: 0.309 +/- 0.099 N/m; E45A/R132T stiffness: 0.225 +/- 0.078 N/m. (TIF) [file ppat.1012537.s005.tif]

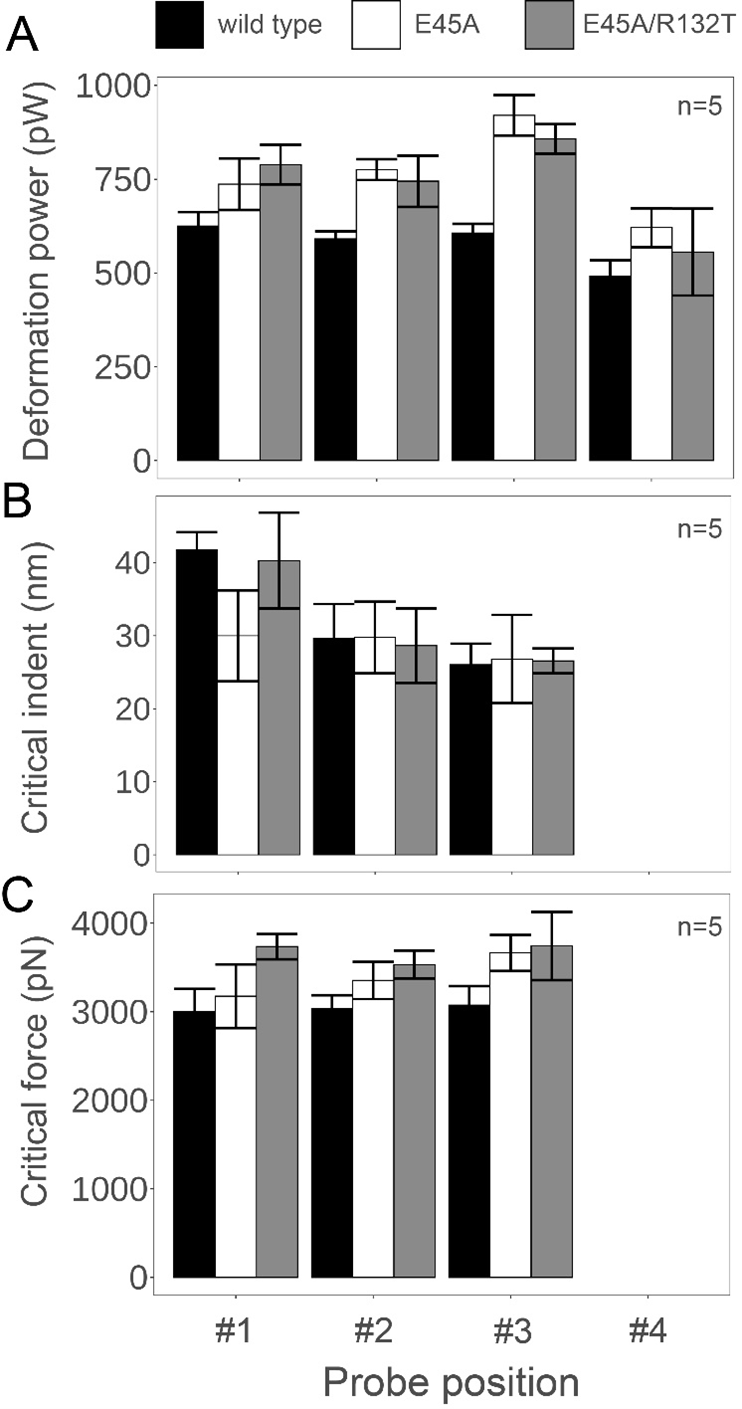

Supplement: S5 Fig — Shown are the values of (A) power, (B) indentation, and (C) force at which the capsid is ruptured. Mean values from n = 5 simulations are shown, with error bars representing the standard deviations. For panels B and C, position #4 values are omitted; failure of the capsid lattice, and thus critical indentation and force values, is not well-defined due to the relatively small height of the sample in the narrow end. (TIF) [file ppat.1012537.s006.tif]

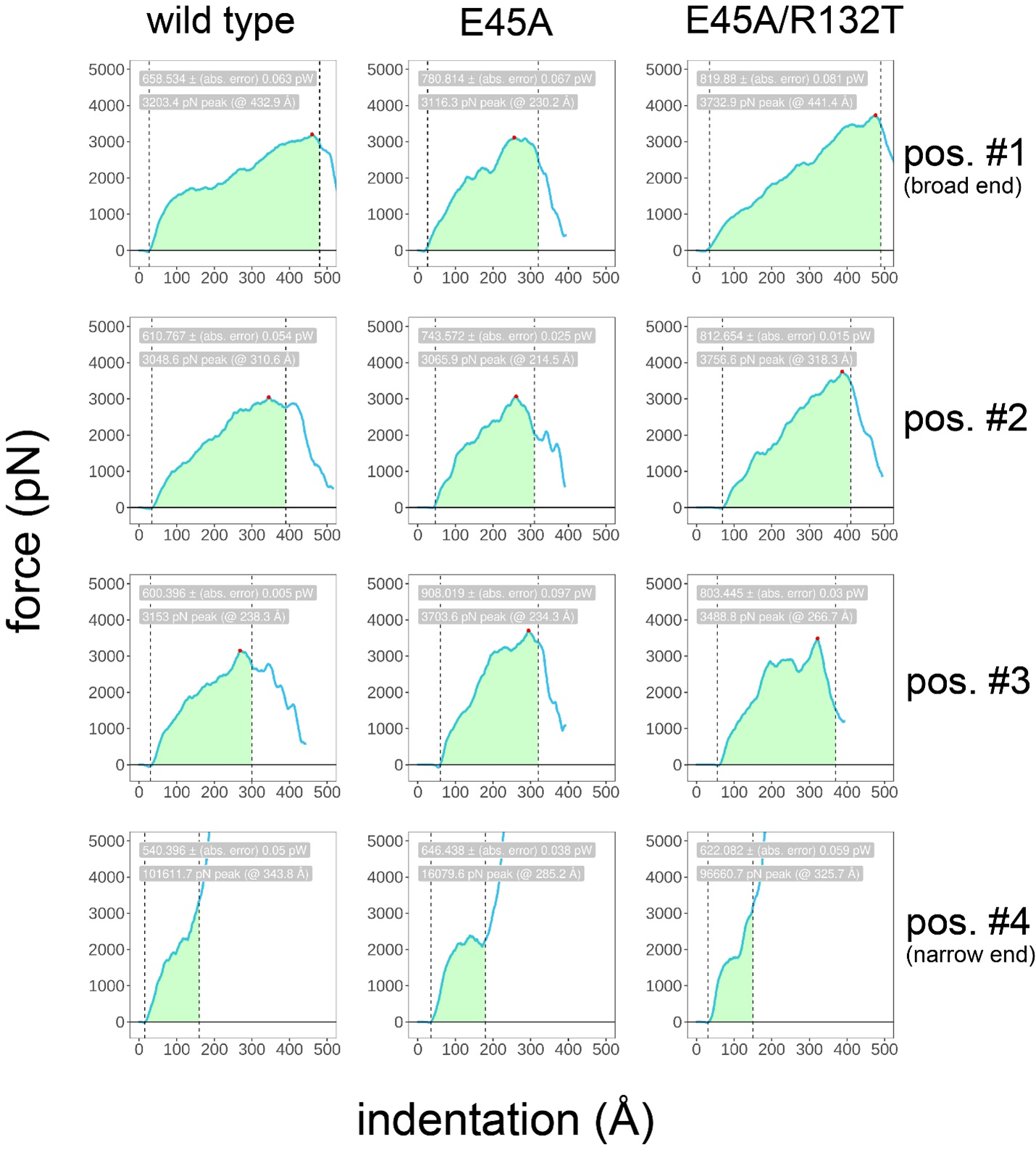

Supplement: S6 Fig — Utilizing a higher probe velocity mimics high-force AFM experiments, where we observed deformation and failure of capsids conferring higher measured forces. In the case of simulations, these events were observed across smaller time intervals. Each column is labeled according to the relevant construct, and each row represents a different probe location along the length of the cone (corresponding to Fig 1B–1E). Each plot is annotated with the power, determined by integration (shown in green), that is summarized in S8 Fig. Integration bounds are shown, the lower of which was set at probe contact and the upper bound is set to encapsulate the yield (critical) force, annotated with a red dot. Power is considered as the time-normalized integral of each curve (green region), presented in units of picowatts with absolute integration error given. For the narrow end probe locations, position four, critical forces and indentation distances are ill-defined due to densification of the capsids. For the latter cases, we set the upper integration bound as immediately prior to densification. This enables the calculation of power but not critical forces or critical indentation values as shown in S4 Fig for the other three probe positions. (TIF) [file ppat.1012537.s007.tif]

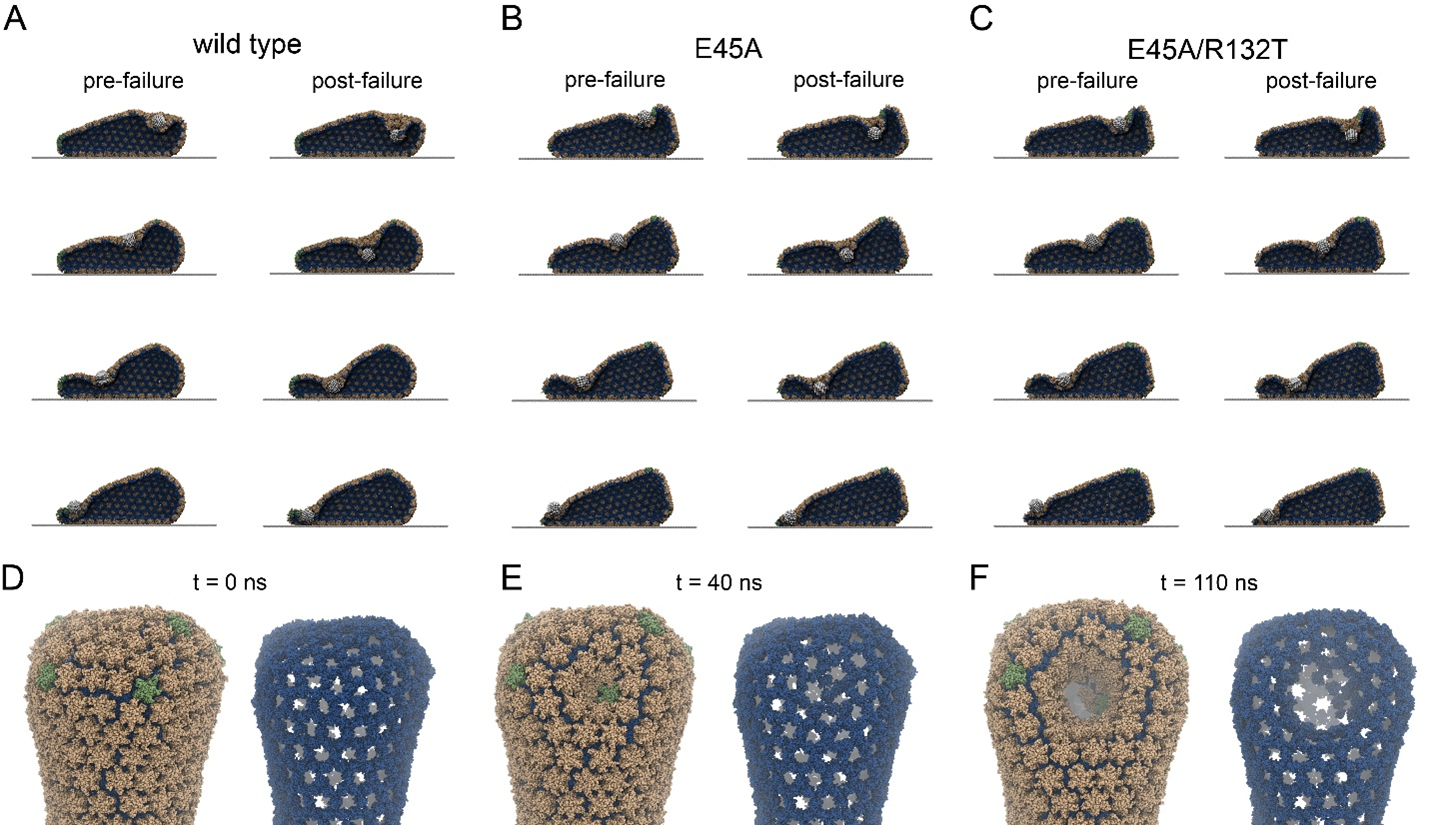

Supplement: S7 Fig — Pre- and post-failure snapshots are shown for: (A) wild type, (B) E45A, and (C) E45A/R132T capsids, with each probe position shown. The velocity of the probe was 312.5 nm/microsecond. Panels D-F show a succession of close-up images of wild type capsid indentation and failure, including a complete view as well as a C-terminal domain only view, to highlight the separation of assembly interfaces. E shows indentation without significant separation of assembly interfaces, slight separation of trimer interfaces is visible. F shows the failure event fully manifest, where a large discontinuity is seen in both the complete and C-terminal domain views. It is worth noting that, as opposed to experimental breakages observed, the features of these lattice failures are highly localized. The latter is owed to the rapid probe velocity employed in simulations, and possibly the geometry employed. S13 Fig shows a view of the undeformed capsid employed in each simulation. (TIF) [file ppat.1012537.s008.tif]

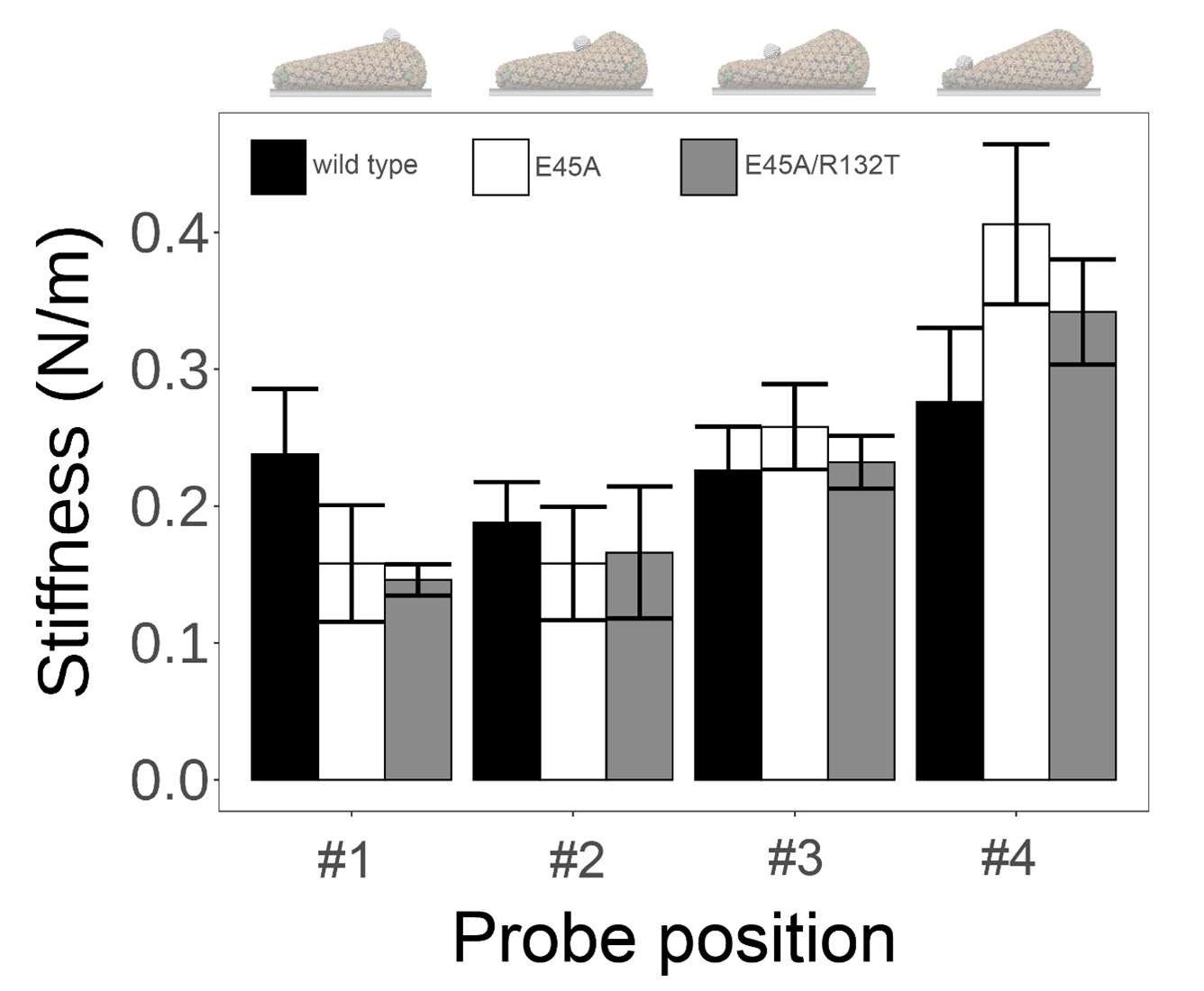

Supplement: S8 Fig — Error bars represent the standard deviation of these five trials. Interestingly, using a significance threshold of 0.05, we see that wild type is significantly stiffer than either mutant at broad end position #1 (p-value = 0.02358 for E45A vs. wild type; p-value = 0.01089 for E45A/R132T vs. wild type). For narrow end position #4, E45A is significantly stiffer than wild type (p-value = 0.006611). For the remaining probe positions, all three constructs confer insignificant differences in stiffness, with p-values > 0.05. This conforms to experimentally derived stiffness trends of wild type, E45A and E45A/R132T capsids. (TIF) [file ppat.1012537.s009.tif]

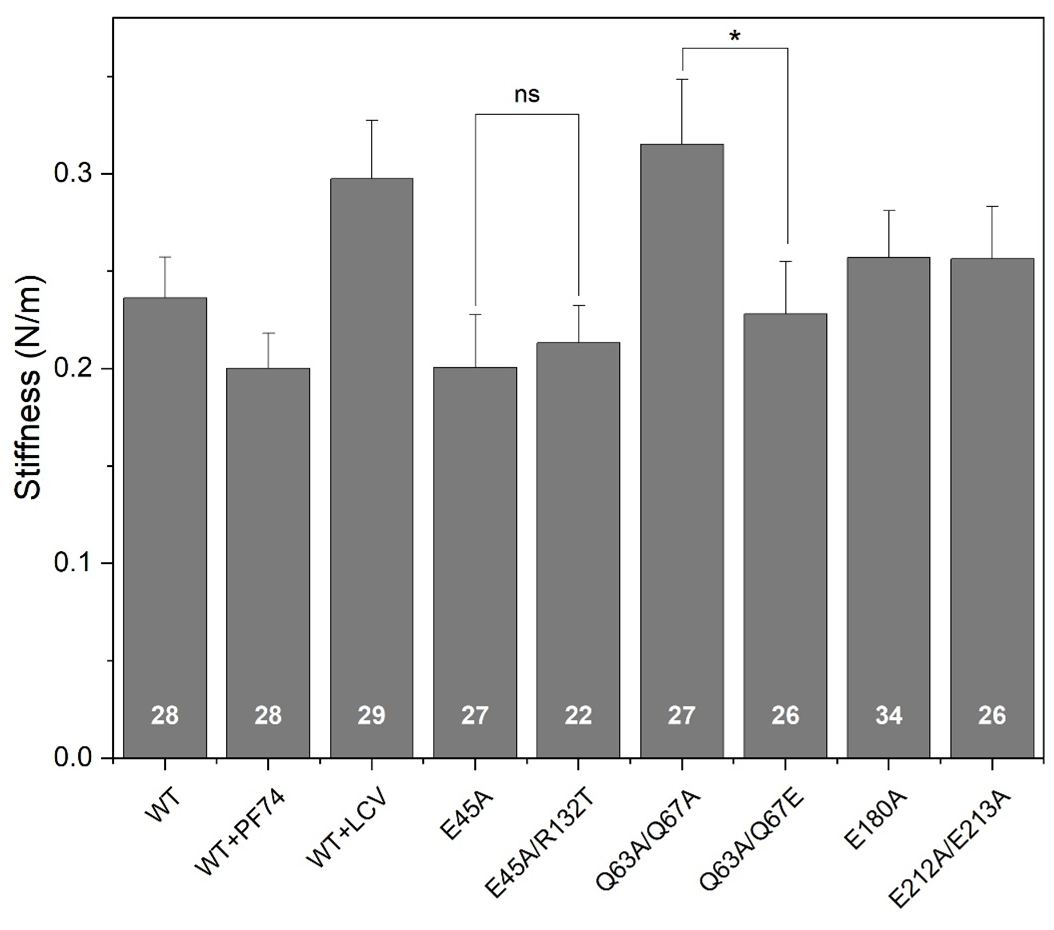

Supplement: S9 Fig — Each stiffness value was calculated as the average of ~480 force–distance curves obtained from individual cores. Measurements were conducted in the presence of inositol hexakisphosphate (IP6; 100 μM). The t-test analysis revealed that differences between the stiffness values of the various samples and WT are statistically significant (p values: <0.001 and <0.05 for WT+IP6+PF74 and E45A+IP6, respectively, <0.0001 for the remaining samples). Error bars represent the standard error of the mean. (TIF) [file ppat.1012537.s010.tif]

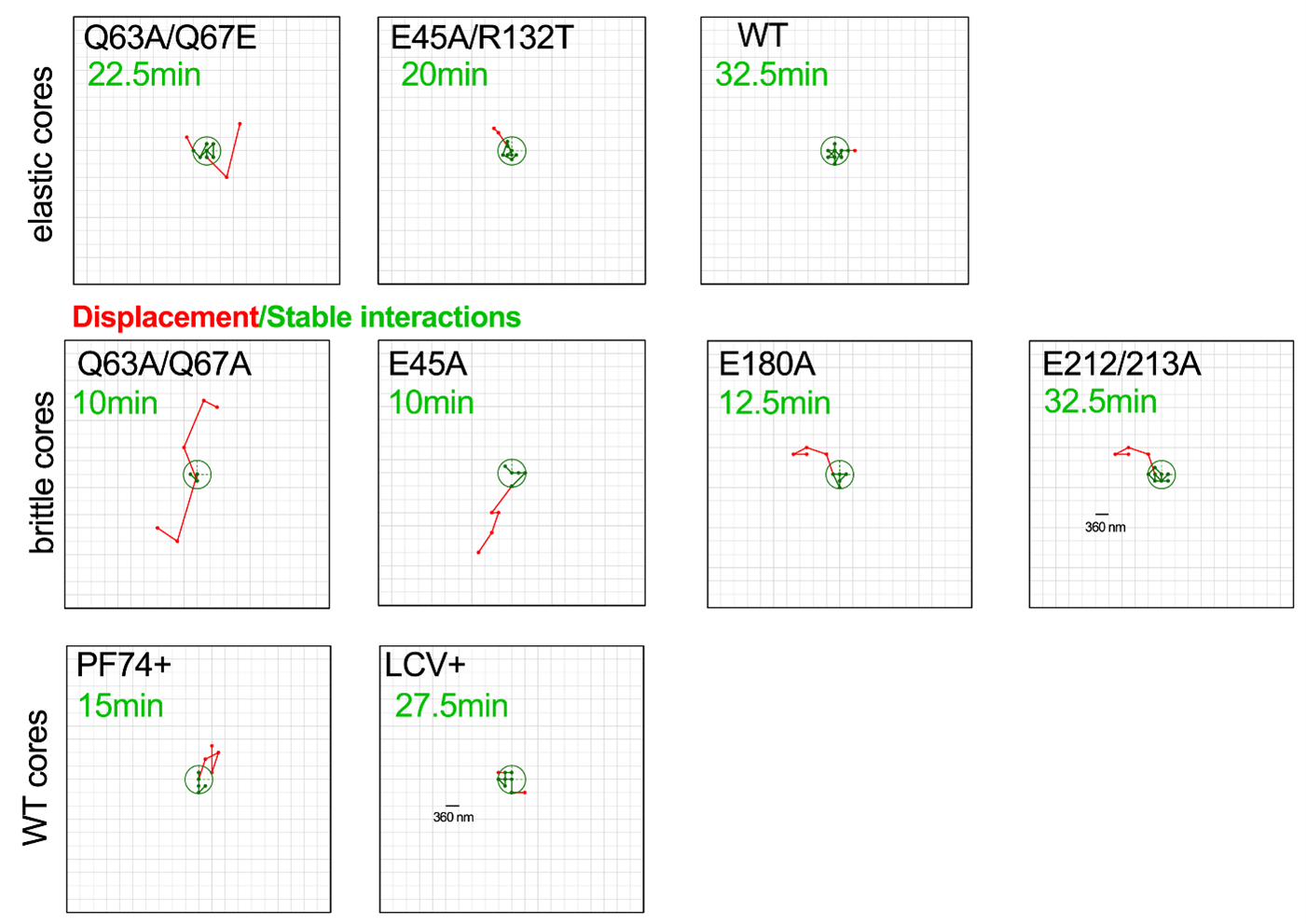

Supplement: S10 Fig — Docking segments (green) and displacements (red) are highlighted. Scale bar is 0.36 μm. (TIF) [file ppat.1012537.s011.tif]

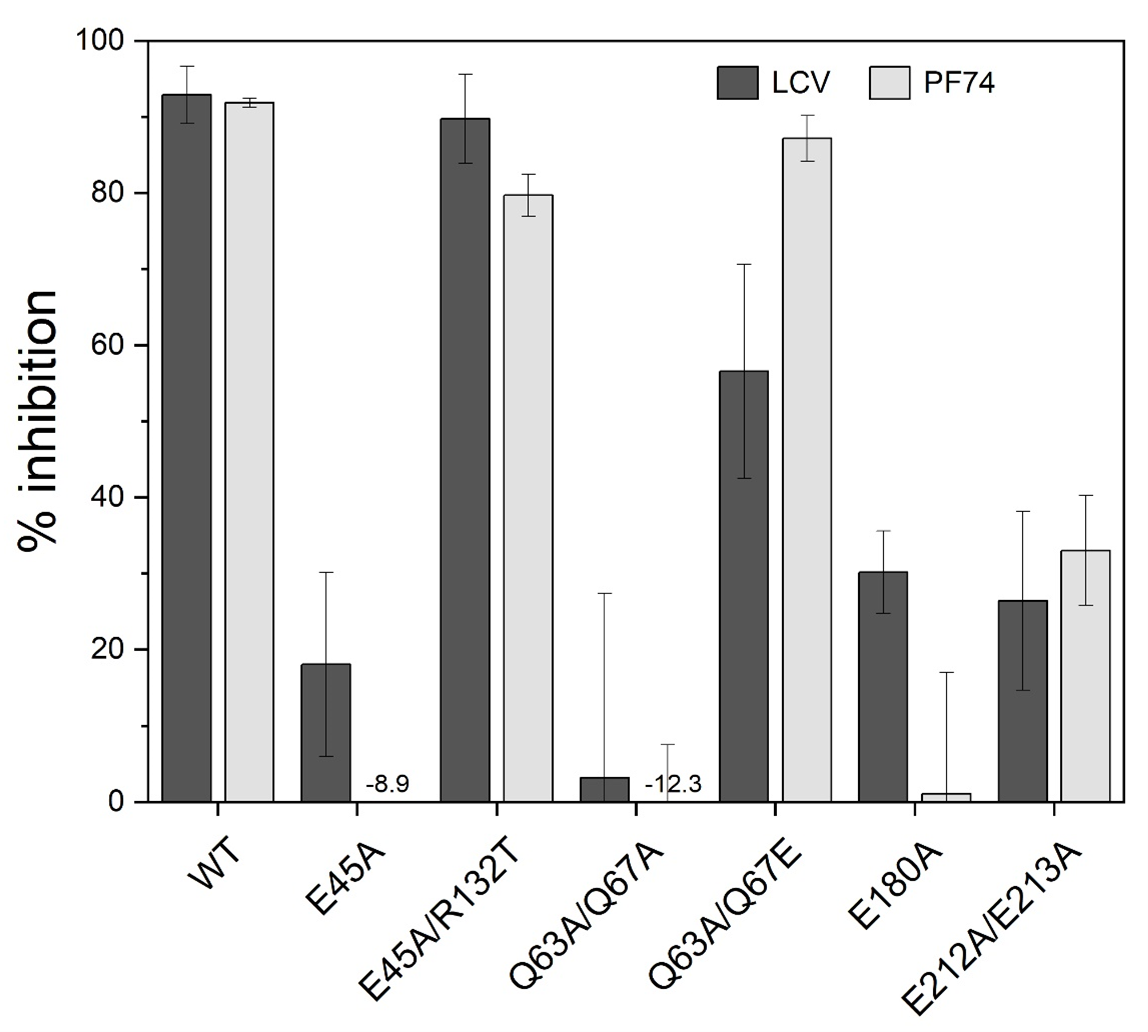

Supplement: S11 Fig — Error bars represent standard deviation of the mean. (TIF) [file ppat.1012537.s012.tif]

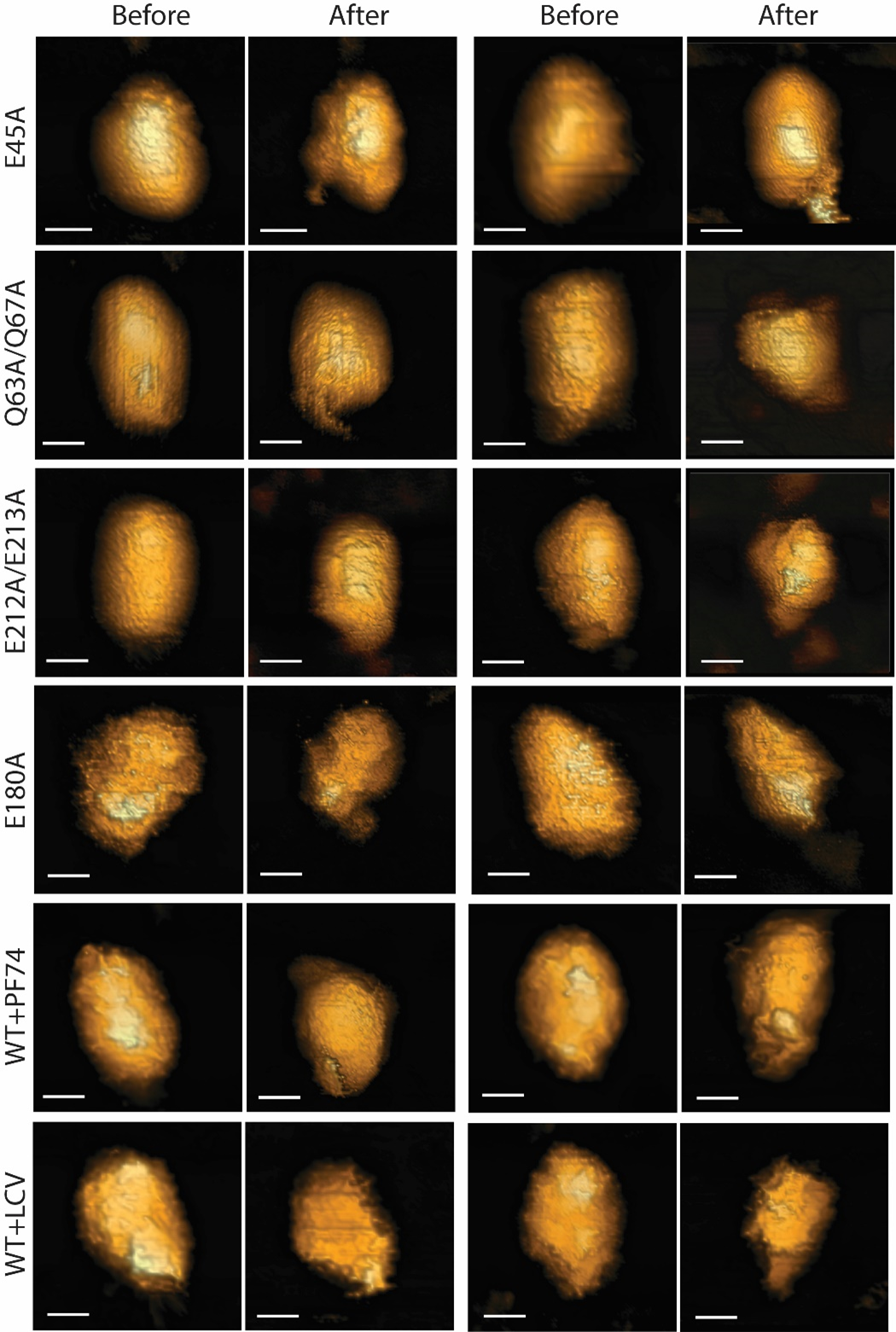

Supplement: S12 Fig — Two representative images for each mutant and WT treated with PF74 or LCV are shown. All images were acquired using the QI mode at a maximal loading force of 300 pN. Scale bars are 60 nm. (TIF) [file ppat.1012537.s013.tif]

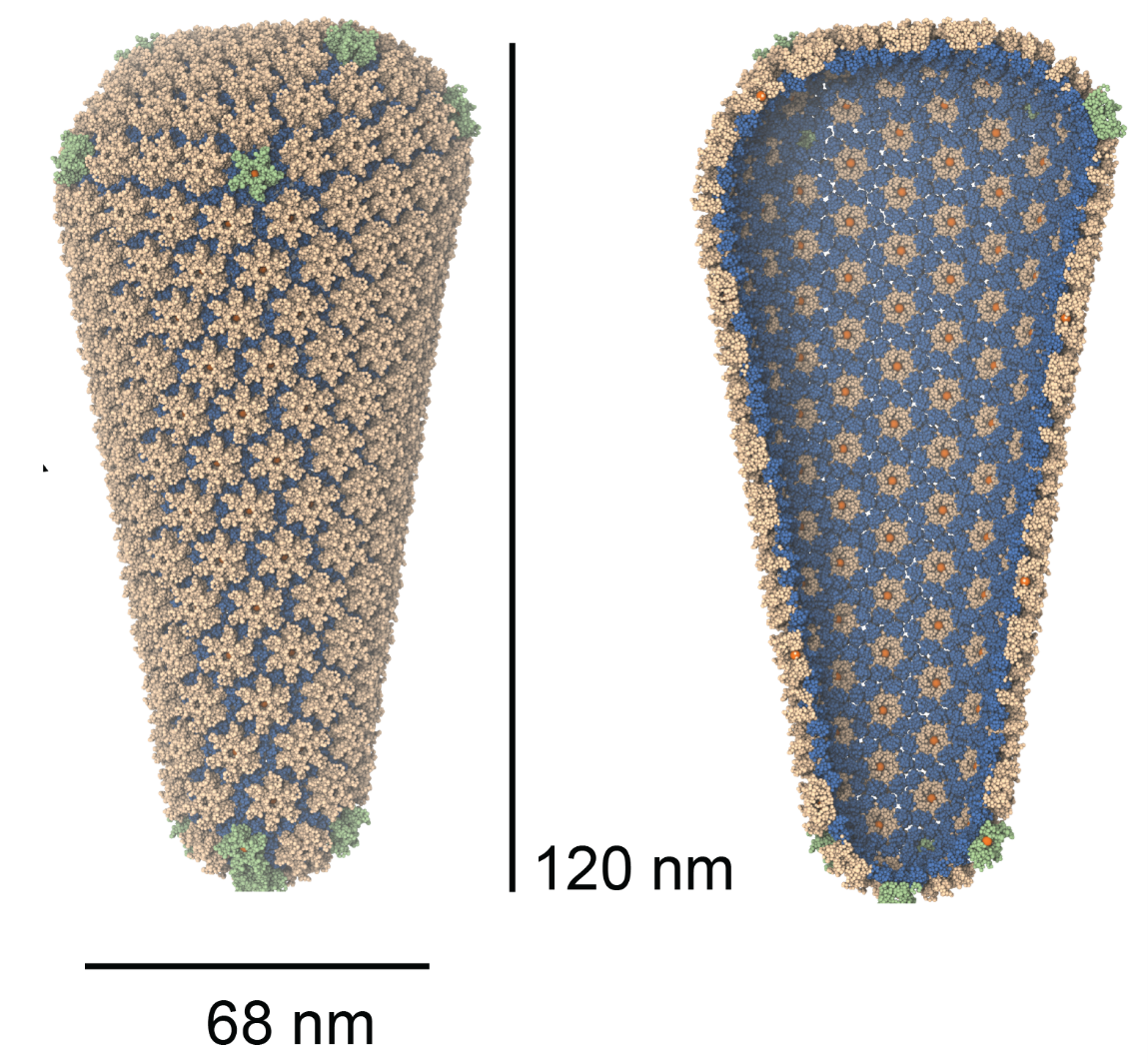

Supplement: S13 Fig — The capsid is shown whole (left) and clipped (right). For the N-terminal domain, capsid hexamers are colored tan and capsid pentamers are colored green. C-terminal domains are colored blue. Orange beads in the centers of each capsomer represent IP6. (TIF) [file ppat.1012537.s014.tif]
