# Supplementary material for: Elasticity of the HIV-1 core facilitates nuclear entry and infection
Source: PLoS Pathog. 2024 Sep 11;20(9):e1012537. doi: 10.1371/journal.ppat.1012537 (PMC11419384; doi:10.1371/journal.ppat.1012537)
Supplement: S1 Text — (DOCX) [file ppat.1012537.s015.docx]

Simulated AFM nanoindentation of the E45A and E45A/R132T mutant capsids revealed a striking increase in stiffness compared to wild type (S4 Fig). Utilizing a 3.125 nm/μs probe velocity, the same as employed for wild type simulations (Fig 1B-E), we obtained significantly higher stiffness values for these mutants. For E45A: 0.309 +/- 0.099 N/m (n=4); for E45A/R132T: 0.225 +/- 0.078 N/m (n=4); and for wild type: 0.077 +/- 0.045 N/m (n=4). While our models revealed a decrease in stiffness of E45A/R132T vs. E45A, the E45A/R132T mutant core remains significantly stiffer than the wild type. The latter result is at odds with experimentally measured stiffness, where wild type is slightly stiffer than E45A and E45A/R132T mutants, and the mutant capsids are similar to one another.

We additionally subjected wild type and mutant capsid models to high-velocity (312.5 nm/μs) AFM simulations (S5 and S6 Figs). These simulations mimic high-force AFM experiments, where forces acting on the simulated AFM probes are higher, and we were able to record and observe failure events and thus determine critical forces and critical indentation values corresponding to failures. These simulations cover smaller timescales, enabling us to perform five repeated trials, amounting to 20 indentation simulations (Table A) for each of the three simulated constructs, with n=5 trials per probe location. Strikingly, time-normalized integration of resulting force-displacement curves showed that E45A and E45A/R132T absorb considerably more power than wild type during indentation (S5A Fig). Moreover, broad end (position #1) deformation showed that E45A failed at significantly shorter indentation distances, whereas E45A/R132T sustained deformations similar to wild type (S5B Fig), despite also sustaining higher critical forces (S5C Fig).

We then computed mean stiffness across the five trials for each construct, treating each probe location independently rather than aggregating all probe positions into a single stiffness value (S8 Fig). We found that the stiffness of each construct deviates significantly only at the highly curved broad and narrows ends (positions #1 and #4, respectively). Similar to the experimentally derived stiffness measurements showing wild type capsids are stiffer than E45A and E45A/R132T mutants, we see that the broad end (position #1) of the wild type capsid is the stiffest of the three constructs tested. Importantly, the flat mid-regions of the conical capsids yield similar stiffness measurements for all three constructs, conforming to experimentally derived trends in stiffness. While the exact distribution of probe locations employed for experimentally derived stiffness measurement is not known precisely, based on considerations of sample surface area we conclude that experimental nanoindentations disproportionately probe the broad end and mid-region of capsids (positions #1, #2 and #3), where wild type capsids confer a stiffer mechanical response and E45A and E45A/R132T mutants show similar responses.

| **Construct** | **Probe velocity (nm/μs)** | **Num. trials** |
| --- | --- | --- |
| Wild type | 3.125 | 3 |
| E45A | 3.125 | 3 |
| E45A/R132T | 3.125 | 3 |
| Wild type | 312.5 | 5 |
| E45A | 312.5 | 5 |
| E45A/R132T | 312.5 | 5 |

Table A. Simulated AFM trials performed. For each construct, a trial is defined as a set of four probe positions, corresponding to Fig 2B-E.

*Description of the mechanical properties used in this study*

In this study, atomic force microscopy was employed to characterize the mechanical properties of HIV-1 cores. The stiffness of these cores was quantified by measuring their compliance to applied forces within the elastic regime. A low force of 1.5 nN was initially applied, during which all samples exhibited elastic behavior. However, most materials possess a maximal force threshold beyond which they no longer maintain elasticity. To assess this threshold, a higher force of 5 nN was applied, and a specific region on the core surface was scanned under this increased force. The capacity of the core to undergo reversible deformation without structural breakage following significant compression was defined as the elasticity of the core. Conversely, cores that experienced structural breakage after compression, particularly nuclear entry-deficient mutants, are described as more brittle.

*Molecular Dynamics*

*Preparation of AFM apparatus: baseplate and probe*

For the base plate, we utilize a rectangular, flat lattice of beads with van der Waals (vdW) radii of 1 nm. Bond distances of 1.5 nm are enforced with force constants of 10 $\mathrm{kcal}\cdot\mathrm{mo}l^{-1}$, lattice regularity and planarity are enforced via 90 and 180 degrees angle terms, respectively, with 100 $\mathrm{kcal}\cdot\mathrm{mo}l^{-1}$ force constants. The beads comprising the base plate are given a Lennard-Jones ε value of -1.5 $\mathrm{kcal}\cdot\mathrm{mo}l^{-1}$, to allow a weak and non-specific anchoring of the capsid to the plate. The AFM tip is modeled with inert beads of vdW radius 1 nm arranged in a cubic lattice, and by excluding all beads that are 6.5 nm away from the lattice’s geometrical center. The resulting probe is a 13 nm diameter sphere where each bead comprising the sphere is bound to its neighbors with an equilibrium distance of 1.5 nm and a force constant of 10 $\mathrm{kcal}\cdot\mathrm{mo}l^{-1}$. Angle terms, either 90 degrees or 180 degrees, are enforced with force constants of 100 $\mathrm{kcal}\cdot\mathrm{mo}l^{-1}$. Four systems were built, where the tip is spaced equidistantly along the capsid’s principal axis of inertia. Capsids were adsorbed for 150 ns prior to AFM simulations.

*Post-processing of force-profile curves*

For plotting, both the raw data and a windowed-average trace, employing a window size of 1,000 points, are shown (Fig 2). Linear fits for estimation of stiffness, in units of $N\cdot m^{-1}$, were employed using the first 3 nm of indentation from each in silico AFM trajectory.
