# Supplementary material for: Elasticity of the HIV-1 core facilitates nuclear entry and infection
Source: PLoS Pathog. 2024 Sep 11;20(9):e1012537. doi: 10.1371/journal.ppat.1012537 (PMC11419384; doi:10.1371/journal.ppat.1012537)
Supplement: S2 Text — (DOCX) [file ppat.1012537.s016.docx]

*Fluorescence Microscopy*

*Virus production*

Fluorescently tagged VSV-G pseudotyped HIV-1 particles was produced as described previously^1^. Briefly, envelope deleted pHIVeGFP CA wild-type (WT) and indicated CA mutants (MT) proviral backbone (2ug), VSV-G envelope (0.5μg) and Vpr-integrase fused to mNeonGreen (INmNG, 0.8μg) was mixed in jetPRIME buffer and 6ul of jetPRIME reagent, and transfected into HEK 293T cells plated at 80% confluency in a 6-well plate. Following a 6h incubation in a CO_2_ incubator, the transfection medium was exchanged for fresh phenol-red minus DMEM complete with antibiotics and 10% FBS. Virus supernatants were collected after an additional 36h of incubation, clarified through a 0.45 μm filter and quantified for RT-activity, aliquoted and stored at -80^◦^C until use.

*Tracking the interactions between HIV-1 capsid mutants and the nuclear pore complex*

In prior work, others including our group used fast-temporal imaging of HIV-1 capsid interactions with the nuclear envelope (NE) in living cells [1,2]. In these experiments, interaction of K203A or E45A mutant cores was negligibly detected, primarily due to the imaging experiment which spanned for only a short time-window (2h). Moreover, it was not feasible to estimate fraction of capsids that dock at the NE during the entry time course of infection. To address these caveats, and to unbiasedly characterize HIV-1 interactions with the NE, we used single HIV-1 tracking to analyze cores that came into contact with the NE (interactions) and their ability to remain immobile at a single site on the NE (docking) over 8h of virus entry steps.

Several of the HIV-1 core tracks detected on the NE corresponded to movement of cores laterally along the NE mask (S10 Fig, red lines). We therefore analyzed segments of each individual track for docking behavior, as defined by the segments of tracks when a single HIV-1 core remained within a 2-pixel (360 nm) radius for 3 or more frames (>7.5 min) (S10 Fig, detection in green circles). These segments were considered as interactions representative of HIV-1 core docking at the NE. The stringent conditions (2-pixel localization of cores for >3 frames) allowed us to robustly define virus docking and distinguish them from transient interactions along the NE, when cores fail to remain at a given location for more than 5 minutes. The total number of docking segments that went in the analysis for elastic capsids was WT = 321, E45A/R132T = 77, and Q63A/Q67E = 71; for the brittle cores was, E45A = 63, Q63A/Q67A = 48, E180A = 77 and E212A/E213A = 523. Of these the fraction of cores showing extended docking (>10min) at the NE was 42.5% for WT, 14.3% for E45A, 19.5% for E45A/R132T, 14.6% for Q63A/Q67A, 19.8% for Q63A/Q67E, 12% for E180A, 28.6% for E212A/E213A and 4.8% for the control envelope deficient bald HIV-1 (no-VSV) cores (Fig 5A and 5B). When compared to the control, the HIV-1 WT and all the MT capsids tested here showed a > 2-fold increase in docking probability (Fig 5B), suggesting their ability to effectively interact with NPCs.

References

1. Francis, A. C. & Melikyan, G. B. Single HIV-1 Imaging Reveals Progression of Infection through CA-Dependent Steps of Docking at the Nuclear Pore, Uncoating, and Nuclear Transport. *Cell Host Microbe* **23**, 536-548 e536, (2018).

2. Burdick, R. C. *et al.* Dynamics and regulation of nuclear import and nuclear movements of HIV-1 complexes. *PLoS Pathog* **13**, e1006570, (2017).
